# Induction of influenza-specific local CD8 T-cells in the respiratory tract after aerosol delivery of vaccine antigen or virus in the Babraham inbred pig

**DOI:** 10.1371/journal.ppat.1007017

**Published:** 2018-05-17

**Authors:** Katie Tungatt, Garry Dolton, Sophie B. Morgan, Meriem Attaf, Anna Fuller, Thomas Whalley, Johanneke D. Hemmink, Emily Porter, Barbara Szomolay, Maria Montoya, John A. Hammond, John J. Miles, David K. Cole, Alain Townsend, Mick Bailey, Pierre J. Rizkallah, Bryan Charleston, Elma Tchilian, Andrew K. Sewell

**Affiliations:** 1 Division of Infection and Immunity, Cardiff University School of Medicine, Cardiff, Wales, United Kingdom; 2 The Pirbright Institute, Pirbright, Woking, United Kingdom; 3 Systems Immunity Research Institute, Cardiff University, Cardiff, Wales, United Kingdom; 4 School of Veterinary Sciences, University of Bristol, Bristol, United Kingdom; 5 James Cook University, Cairns, Australia; 6 Weatherall Institute of Molecular Medicine, University of Oxford, Oxford, United Kingdom; University of Iowa, UNITED STATES

## Abstract

There is increasing evidence that induction of local immune responses is a key component of effective vaccines. For respiratory pathogens, for example tuberculosis and influenza, aerosol delivery is being actively explored as a method to administer vaccine antigens. Current animal models used to study respiratory pathogens suffer from anatomical disparity with humans. The pig is a natural and important host of influenza viruses and is physiologically more comparable to humans than other animal models in terms of size, respiratory tract biology and volume. It may also be an important vector in the birds to human infection cycle. A major drawback of the current pig model is the inability to analyze antigen-specific CD8+ T-cell responses, which are critical to respiratory immunity. Here we address this knowledge gap using an established in-bred pig model with a high degree of genetic identity between individuals, including the MHC (Swine Leukocyte Antigen (SLA)) locus. We developed a toolset that included long-term *in vitro* pig T-cell culture and cloning and identification of novel immunodominant influenza-derived T-cell epitopes. We also generated structures of the two SLA class I molecules found in these animals presenting the immunodominant epitopes. These structures allowed definition of the primary anchor points for epitopes in the SLA binding groove and established SLA binding motifs that were used to successfully predict other influenza-derived peptide sequences capable of stimulating T-cells. Peptide-SLA tetramers were constructed and used to track influenza-specific T-cells *ex vivo* in blood, the lungs and draining lymph nodes. Aerosol immunization with attenuated single cycle influenza viruses (S-FLU) induced large numbers of CD8+ T-cells specific for conserved NP peptides in the respiratory tract. Collectively, these data substantially increase the utility of pigs as an effective model for studying protective local cellular immunity against respiratory pathogens.

## Author summary

Influenza virus infection in pigs represents a significant problem to industry and also carries substantial risks to human health. Pigs can be infected with both bird and human forms of influenza where these viruses can mix with swine influenza viruses to generate new pandemic strains that can spread quickly and kill many millions of people across the globe. To date, the study of immunology and vaccination against flu in pigs has been hampered by a lack of suitable tools and reagents. Here, we have built a complete molecular toolset that allows such study. These tools could also be applied to other important infections in pigs such as foot-and-mouth disease and the normally fatal African Swine Fever virus. Finally, pigs are set to become an important model organism for study of influenza A virus infection. Here, we make use of a new research toolset to study a Broadly Protective Influenza Vaccine (BPIV) candidate, S-FLU, which could offer protection against all influenza A viruses. These new tools have been used to demonstrate the induction of large numbers of antigen specific CD8+ T cells to conserved NP epitopes in the respiratory tract after aerosol immunization.

## Introduction

Infection with influenza A virus is a major cause of human morbidity and mortality globally. Influenza is highly infectious, constantly mutating and infects a range of host species including humans, pigs and birds. Human influenza infection places a large burden on health care resources and has been estimated to cost >$85 billion annually in the United States alone [1]. Vaccination strategies in humans for seasonal influenza infection require annual reformulation of the vaccine to combat the constantly changing virus. Seasonal immunization induces antibodies, predominantly against the viral protein haemagglutinin, which neutralize the immunizing strain very effectively, but escape variants rapidly emerge and are responsible for antigenic drift. Therefore development of a broadly protective influenza vaccine (BPIV) would represent a major advance [2,3]. Individuals previously infected by one influenza subtype often show reduced disease severity following subsequent infection with a different influenza subtype in the absence of neutralising antibodies to the new strain. This phenomenon is known as ‘heterotypic immunity’ [4] and experimental studies indicate that T-cell responses, particularly CD8+ T-cells that recognize conserved epitopes of internal viral proteins, are key to limiting the severity of disease following repeated influenza infections [5–9].

Influenza is endemic in the global pig population and domesticated animals might be pivotal hosts in generating highly dangerous pandemic strains in future [10–12]. Three strains of swine influenza virus (SwIV), H1N1, H1N2 and H3N2 constantly circulate in pigs. However, pigs can be infected with both avian and human influenza strains, allowing genetic reassortment. This antigenic shift can produce new and highly virulent influenza strains to which humans are immunologically naïve [10]. Therefore, it is believed that pigs may serve as ‘mixing vessels’ for the generation of human-avian influenza A virus reassortments, similar to those responsible for the Asian H2N2 and Hong Kong H3N2 pandemics in 1957 and 1968, respectively [11]. Transmission of Influenza viruses between humans and pigs has also been well documented globally; between 2009–2011 one study identified almost 50 transmission events of pandemic H1N1, along with over 20 transmissions of H1 and H3 viruses since 1990 [13]. Indeed, more recent analyses suggest that the 1918 HIN1 pandemic IAV strain may have transferred from human to swine rather than the other way around [14,15]. The ample capacity for spillover and spillback of IAV infection between humans, swine, birds and other mammalian species makes it difficult to be certain of the precise origins of historical IAV pandemic strains [14].

In addition to being a potential source of zoonotic influenza viruses, the pig is an optimal model of human influenza infection since both swine and human influenza strains replicate to similar levels in the upper and lower respiratory tract of pigs, exhibit similar patterns of viral shedding and exhibit comparable distribution of sialic acid receptors [16–20]. Consequently, understanding influenza infection in pigs has enormous potential for combating and controlling this most serious of zoonotic threat to global human health.

Influenza infection in pigs also imposes a significant economic burden of its own. Although SwIV typically causes mild disease, losses can be incurred due to reduced weight gain, suboptimal reproductive performance and secondary infections [2]. Pig farming represents a significant sector of the global livestock industry, with animal numbers estimated to be over 980 million worldwide in 2016 [21]. Effective vaccination strategies and biosecurity practices would help eliminate the financial burden of SwIV and improve animal welfare as well as helping to relieve poverty in developing countries.

Despite the potential role of pigs as a source of new influenza viruses, the immune response to SwIV in pigs has been understudied. This deficiency has arisen, at least in part, due to a lack of research tools to study T-cell responses in pigs and the inability to culture pig T-cells long term *in vitro*. Recent studies in outbred pigs used *in silico* prediction algorithms to identify SwIV epitopes and peptide-SLA (pSLA) tetramers have been produced [22,23]. Although pSLA tetramers have been tested for T-cell binding in hyper-immunized or repeatedly infected animals, they have not so far been exploited to understand the biology of practically useful vaccination regimes or infection. For study, we selected the Babraham large white, inbred pig that is 85% identical by genome wide SNP analysis [24]. The matching of SLA class I and II alleles between individual animals makes the Babraham pigs invaluable for immunological studies by allowing adoptive transfer of immune cells between individuals. Importantly, the principles of replacement, reduction and refinement of animal experiments are supported by the use of these animals because fewer numbers of animals per group can be used for immunological studies compared with outbred pigs.

Here, we aimed to bring the study of T-cells in the Babraham pig up to the level of that available for influenza study in humans and experimental mice. This goal required that we establish pig T-cell *in vitro* culture and cloning for the first time. Successful establishment of conditions for pig T-cell culture enabled us to define influenza-derived, immunodominant CD8β+ cytotoxic T-cell epitopes in the Babraham pig. Soluble peptide-SLA (pSLA) complexes were generated allowing construction of fluorochrome-conjugated pSLA tetramers and determination of the molecular structures of each Babraham SLA-I molecule complexed with different influenza epitopes. The latter enabled the determination of which amino acids within the peptide acted as primary SLA anchors, from which the SLA binding motif for each of the SLA-I alleles was generated. Peptide-SLA tetramers for dominant epitopes were used to study antigen-specific T-cell responses to the BPIV candidate S-FLU [25–27] or SwIV infection and demonstrated the magnitude and dynamics of local lung immune responses.

## Materials and methods

**Fig 1:**
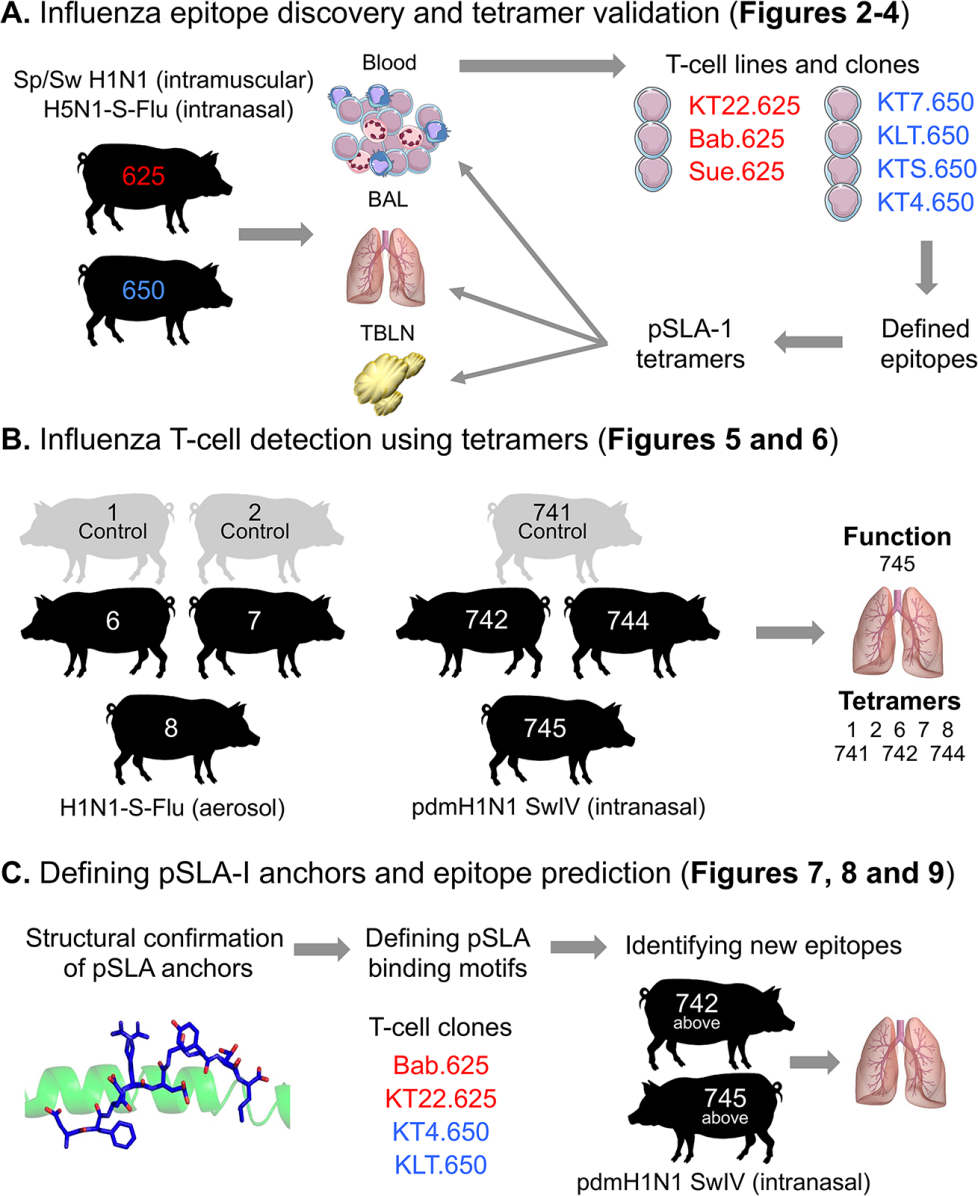
Study overview. The inbred Babraham pig was used throughout this study for vaccination and infection, with each pig assigned an identifying number, shown here within each silhouette. (**A**) Pigs 625 (red) and 650 (blue) were vaccinated intranasally and intramuscularly as depicted. Blood, bronchoalveolar lavage (BAL) and tracheobronchial lymph nodes (TBLNs) were harvested, with peripheral blood mononuclear cells (PBMCs) purified from blood and single suspensions from BAL and TBLNs generated for experiments. Overlapping peptides from the NP of PR8 were used to create T-cell lines (**Fig 2**) and T-cell clones (named and shown in red or blue text) (**Fig 3**). The red clones came from pig 625 (red) and the blue from pig 650 (blue). The clones were used to define minimal NP peptides, which were subsequently refolded with SLA-1*14:02 or SLA-2*11:04 to create pSLA-I tetramers. The tetramers were used to stain the clones (**Fig 3**) and harvested tissues from pigs 625 and 650 (**Fig 4**). (**B**) The BAL from pigs vaccinated or infected intranasally with influenza, as shown, were stained with the tetramers from A (**Figs 5 and 6**). The BAL from pig 745 was used for *ex vivo* ELISPOTS. (**C**) SLA-1*14:02 or SLA-2*11:04 were refolded with the epitopes defined in A to confirm peptide anchor residues (**Fig 7**). T-cell clones from A were used to define a SLA-1*14:02 or SLA-2*11:04 peptide anchor binding motif (**Fig 8**), which were then used to predict other influenza epitopes, tested using BAL from the two pigs shown (**Fig 9**).

An overview of this study including the Babraham pigs used for each experiment is shown in **Fig 1**.

### Babraham pig and SLA-I alleles

All experiments were performed on the Babraham large white inbred pig line and conducted at either the Pirbright Institute or the University of Bristol. All Babraham pigs were confirmed influenza free by screening for the absence of influenza infection by matrix gene real time RT-PCR [28], and antibody-free status was confirmed by haemagglutination inhibition using 4 SwIV antigens. The body weight and age of all Babraham pigs used for this study are detailed in **S1 Table**. At the inception of this study the Babraham pig colony was maintained with a minimal number of breeding animals: as such, historic samples were used and new pigs enlisted when they became available. As a result, the pigs utilized ranged in age, size and sex (**S1 Table**). Also, as the pigs were being used for parallel, yet to be published, studies the mode of influenza exposure varied depending on the cohort. To identify the Babraham pig SLA-1 and SLA-2 alleles a degenerate primer set was designed using all the porcine SLA class I sequences available on IPD-MHC (http://www.ebi.ac.uk/ipd/mhc) (SLA-1S 5’GACACGCAGTTCGTGHGGTTC3’; SLA-1AS 5’GCTGCACMTGGCAGGTGTAGC3’). These primers were used to amplify the class I alleles from cDNA available from all the pigs used in this study (**S1 Table**). These were cloned and Sanger sequenced to identify the Babraham pig SLA alleles and confirm that there was no polymorphism between the individuals used. The SLA-I alleles were confirmed as SLA-1*14:02 [Acc. No. IPD0006136] (formerly known as SLA-1*es11) and SLA-2*11:04 [Acc. No. IPD0006176] (formerly known as SLA-2*es22).

### Ethics statement

Animal experimentation was approved by the Pirbright Institute Ethical Review Board under the authority of a Home Office project licence (70/7505) in accordance with UK Home Office Guidance on the Operation of the Animals (Scientific Procedures) Act 1986 and associated guidelines.

### Influenza viruses

The design and production of the BPIV candidate, S-FLU, have been previously described [25]. S-FLU is a non-pathogenic, single cycle, pseudotyped influenza virus, which has its HA-signal sequence suppressed preventing it from replicating within the host [25]. S-FLU virus expresses the core proteins from the PR8 strain [influenza (A/PuertoRico/8/1934(H1N1))] and is coated with a chosen haemagglutinin (H), using a transfected complementing cell line, to enable initial host cell entry. Two vaccine formulations were used, H1N1- and H5N1- S-FLU, that are described in detail in [25,26]. H5N1 S-FLU was coated with the H5 HA of the avian influenza virus A/Vietnam/1203/2004 (clade 1) with the polybasic site removed and replaced with a trypsin cleavage site and encodes eGFP replacing the H1 coding sequence and the N1 NA from A/PR/8/1934, and is designated formally as [S-eGFP/N1(PR8)].H5(VN1203). H1N1 S-FLU, designated [S-eGFP/N1(Eng195)].H1(Eng195)], was coated with the H1 HA, and encodes the N1 neuraminidase (N) from A/England/195/2009. Inactivated virus of the H1N1 strain [A/Swine/Spain/SF11131/2007] (Sp/Sw) was also used for immunization. The swine isolate of A/Swine/Eng/1353/09(pdmH1N1) was used for experimental infection. Sequence conservation of the nucleoprotein (NP) peptides defined in this study between the different strains of influenza used can be found in **S2 Table**.

### Tissue culture reagents

For the culture of T-cells, pig serum was isolated from the clotted blood of non-Babraham pedigree and crossbreed pigs. The blood was collected as a by-product from the University of Bristol Veterinary School abattoir, adhering to EU regulated methods. Up to 300 mL of whole blood was collected per animal and the clotted blood centrifuged (700 g for 20 min increments) to aid the harvesting of serum, which was subsequently mixed from multiple pigs and heat-inactivated at 56°C for 1 h before being frozen at -20°C for long-term storage. Thirty non-Babraham pigs were used to generate the serum used during this study with 25–100 mL of serum harvested per pig. Pig serum was used without influenza testing; with each batch giving very similar performance for T-cell line generation and functional assays, which were performed with media/serum alone controls. Although the 56°C treatment of serum for 1 h was not used specifically to inactivate influenza, it has been shown that pasteurization of egg products at 56°C reduced inoculated avian influenza virus titre by >90% in <2 min [29].

Pig serum and all other media components were filtered through a 0.22 μm membrane prior to addition to the medium. The following media were used in this study: R0 medium (RPMI-1640 Medium supplemented with 2 mM L-glutamine, 100 U/mL Penicillin and 100 μg/mL Streptomycin (all Life Technologies)); R5 medium (R0 supplemented with 5% pig serum); R10 medium (R0 supplemented with 10% heat-inactivated foetal bovine serum (FBS) (Life Technologies)); priming medium (R0 supplemented with 10% pig serum, 10 mM HEPES buffer, 0.5X MEM amino acids, 1 mM Sodium Pyruvate (all Life Technologies), 50 μM 2-Mercaptoethanol and human IL-2 (Aldesleukin, brand name Proleukin, Prometheus); expansion medium (as for priming media but supplemented with swine IL-15 (Kingfisher Biotech). All cells were cultured at 37°C in humid 5% CO_2_ incubators.

### Cryopreservation of cells

T-cell clones and lines, kidney and tissue derived cells were cryopreserved in freezing media consisting of 90% FBS and 10% DMSO. This was performed in 1–1.5 mL cryovials using a CoolCell (Biocision) or Mr Frosty (Nalgene) controlled-rate freezing device placed at -80°C. Long term storage of cells was in liquid nitrogen. Cells were defrosted rapidly at 37°C and resuspended in R10. Tissue derived cells were treated with 10–50 μg/mL of DNAse (Roche) for 10–15 min before being washed ready for assays.

### Babraham kidney epithelial cell line

Healthy renal cortex tissue taken from a Babraham pig was cut into small pieces and digested in RPMI-1640 (Life Technologies) supplemented with 20% Dispase (Sigma Aldrich), 100 U/mL Penicillin, 100 μg/mL Streptomycin and 0.5 μg/mL of Amphotericin B for 2 h at 37°C. Digested tissue was passed through a 70 μm filter, followed by centrifugation at 400 g for 5 min, washing twice with PBS and resuspended in R10 medium. Cells were cultured in a flask overnight and non-adherent cells removed and fresh R10 added. Once confluent, the medium was changed to D10/F12 (as for R10 using DMEM/F12 media (Life Technologies) and 2 mM L-glutamine and 10 mM HEPES) and the cells split 1:5 into petri dishes. Single cell colonies with epithelial morphology were selected to eliminate fibroblast contamination. The kidney cell lines was grown as adherent cells in tissue culture flasks with D10/F12 medium, passaged once a week or when they reached 80–90% confluence, using TrypLE express (ThermoFisher Scientific) to detach the cells. Cells were split 1:2 to 1:20 at each passage.

### Tissue harvest and preparation

Peripheral blood mononuclear cells (PBMCs) were extracted from blood by conventional density gradient centrifugation using Histopaque (Sigma) or Lymphoprep (Axis Shields) at 800 g for 30 min, followed by harvesting the interface, washing, and then lysing the red blood cells using standard ammonium chloride solution. For harvesting bronchoalveolar lavage cells (BAL) the right lobe of the lung was infused with 150 mL of virus transport media comprising culture medium 199 (Sigma-Aldrich) supplemented with 2 mM HEPES, 0.035% sodium bicarbonate, 0.5% BSA, 100 U/mL penicillin, 100 μg/mL streptomycin and 100 U/mL nystatin. Typically, 100 mL was retrieved and then centrifuged at 800 g for 15 min, the cells washed in PBS and passed through a 700 μm filter (25). The composition of the BAL was on average 6–7% CD3^+^ (**S3 Table**) and the remaining being macrophages, with total cell numbers ranging between 4x10^8^ and 1x10^9^. Tracheobronchial lymph nodes (TBLN) were dissected and manually disrupted using the plunger of a 10 mL syringe, passed through a 70 μm filter, washed and red blood cells lysed [27]. Single cell suspensions from all tissue types were stored by cryopreservation for later use.

### T-cell lines from Babraham pigs 625 and 650

#### Vaccination

In order to induce a maximal immune response, Babraham pigs 625 and 650 were immunized simultaneously with 8x10^7^ TCID_50_ H5N1 S-FLU intranasally using a mucosal atomization device (MAD300, Wolfe Tory Medical) and with 2x10^7^ TCID_50_ inactivated H1N1 [A/Swine/Spain/SF11131/2007] (Sp/Sw) with montanide adjuvant intramuscularly. The pigs received an identical booster immunization 25 days later. Pigs were euthanized (stunning with exsanguination) at day 38 (day 13 post boost) and blood, BAL and TBLNs harvested.

#### Peptides

Overlapping peptides were designed to span the entire sequence of the NP from PR8 of S-FLU using a Peptide Library Design and Calculator Webtool (Sigma Aldrich) (**S4 Table**). We designed 81 peptides of 18 amino acids in length with a 12 amino acid overlap region based on information from studies of HIV [30] and influenza [31], and overlapping peptide pools available from biotechnology companies (PepTivators from Miltenyi Biotec and PepMixes from JPT). The biophysical property of each 18mer peptide was examined and the length adjusted by deletion or addition of amino acids from neighbouring peptides to increase the likelihood of them solubilizing in aqueous solution, as precipitation would reduce the concentration of a peptide once in media. Peptides at this stage of the study were synthesized to >70% purity (GLS Biochem Shanghai Limited), rather than the standard crude purity of >40%, to help avoid the possibility of priming T-cells to ‘contaminant’ peptides. Additionally, >70% peptides offer some financial saving when compared to >90% purity peptides, especially when multiple peptides are required. Peptides were reconstituted in DMSO then stored at -80°C and 1 mM working stocks made in R0 medium and stored at -20°C.

#### T-cell line generation

The generation of NP peptide specific T-cell lines involved the separation of cytotoxic T-cells as defined by CD8β expression [32] from PBMCs, with CD8β^neg^ cells from the same pig used as peptide presenting cells. PBMCs were incubated in 50 μL per 3x10^6^ cells with 10 μg/mL mouse anti-pig CD8β (clone PG164A, Kingfisher Biotech) then 1 μg/mL anti-mouse Ig-PE (polyclonal; BD Biosciences), both on ice for 20 min, before using anti-PE microbeads according to the manufacturer’s instructions (Miltenyi Biotec). The CD8β^neg^ cells were incubated at 37°C for 1 h with either DMSO as a control or peptide pools A, B, C or D with each pool containing different overlapping peptides from the NP of S-FLU (PR8) (**S4 Table**). Each peptide was incubated with the CD8β^neg^ cells at 3 μM, after which the cells were irradiated at 3000–3100 rad. The purified CD8β (50,000) cells were cultured with 200,000 CD8β^neg^ cells in priming medium in 96 well round-bottomed tissue culture plates (Greiner) and fed bi-weekly with priming medium for two weeks, before being tested for responses to the peptides that they were primed with.

#### Functional analysis of T-cell lines

Intracellular cytokine staining was used to assess the reactivity of primed T-cell lines for overlapping peptides from NP. T-cell lines were washed by centrifugation at 400 g for 5 min in R0 medium then incubated at 37°C for 5 h with 2 μM peptide(s) in 100 μL (96 well round-bottom culture plates) of R5 medium containing GolgiStop and GolgiPlug (both BD Biosciences), as per the manufacturer's instructions. Cells were labeled with LIVE/DEAD Violet stain (1:40 dilution in PBS then 2 μL per stain in 50 μL) (Life Technologies) at room temperature (RT) for 5 min and mouse anti-pig CD8β (PG164A) and anti-mouse Ig-PE as above. Cells were then incubated with BD Cytofix/Cytoperm solution (BD Biosciences) on ice for 20 min, the proceeding wash steps were then performed with 10% BD PermWash buffer (BD Biosciences). Cells then received 2.4 μg/mL (0.12 μg/sample) anti-human TNF PerCP (cross-reactive with pigs; MAb11; Biolegend) on ice for 20 min. All data were acquired on a BD FACSCanto II flow cytometer using FACSDiva software and data analyzed using FlowJo version 10.0 (TreeStar Inc., U.S.).

### T-cell clones from Babraham pigs 625 and 650

#### Cloning and culture

T-cell clones were procured by limiting dilution directly from NP peptide specific T-cell lines, or by firstly enriching for peptide reactive T-cells. For the latter, T-cell lines were washed in R0 medium and rested in R5 medium overnight. Subsequently, the T-cell lines were incubated with 30 μM of TNFα processing inhibitor (TAPI)-0, anti-TNF antibody and desired peptides for 4–5 h followed by staining for dead cells (**S1 Fig**). The cells were then sorted based on TNF staining using a BD FACS Aria (Central Biotechnology Services, Cardiff University, UK) and cultured overnight in expansion media ready for cloning. For cloning, 0.3–1 T-cells were plated per well of a 96 well round-bottomed plate with 50,000–100,000 irradiated (3000–3100 rad) PBMCs mixed from three non-Babraham pedigree or crossbred pigs (as for the serum above). The irradiated PBMCs from these pigs created an allogeneic environment for the Babraham pig T-cells to grow, with the only criteria being that they were from pig breeds other than the Babraham and therefore likely to express allo-MHC. PBMCs from these pigs were purified from blood using conventional density layer centrifugation and stored as above. The T-cells and irradiated PBMCs were cultured in 50–100 μL per well of T-cell expansion media with 1–3 μg/mL of phytohaemagluttinin (PHA) (Alere, Thermo Scientific, U.S.).

#### T-cell clone screening

Each clone was incubated overnight at 37°C with either peptide(s), media alone or 10 μg/mL PHA (positive control) with all conditions performed in duplicate. The following day, cells were pelleted by centrifugation (400 g for 5 min) and culture supernatants harvested for measurement of MIP-1β by ELISA in half-well flat bottom microplates (Corning Costar) as per the manufacturer's protocol (DuoSets, R&D Systems). However, the following antibodies and protein standards were used: 1.5 μg/mL anti-swine macrophage inflammatory protein (MIP)-1β polyclonal Ab, swine MIP-1β recombinant protein and 0.4 μg/mL biotinylated anti-swine MIP-1β polyclonal Ab (all Kingfisher Biotech). We have previously found that MIP-1β provides an extremely sensitive readout for human [33] and mouse CD8+ T-cells so it was used here for pig T-cells. Clones that tested positive for a pool of peptides were then tested against individual peptides using the same approach as detailed above. Figures were produced in GraphPad Prism version 5.03 (GraphPad Software). The T-cell clones were then used to define minimal NP peptide epitopes.

### Defining minimal epitopes using T-cell clones from Babraham pigs 625 and 650

T-cell clones from pigs 625 (Sue.625 and KT22.625) and 650 (KT7.650 and KTS.650) were incubated with decreasing concentrations of truncated peptides and activation assessed by MIP-1β ELISA, using 7,000–25,000 cells per well. Truncations were performed at the amino- and carboxyl- terminus of the overlap region common to neighbouring peptides. If truncations of the overlap region led to a decrease in T-cell activation, amino acid residues present in one of the neighbouring peptides, but not in the overlap region, were also tested to define the minimal epitope. Peptides were synthesized to >40% purity (GL Biochem Shanghai Limited) as it allowed rapid synthesis of the peptides and subsequent screening of clones that were growing in culture. The original overlapping peptides of >70% purity were tested alongside the >40% peptides to ensure similar reactivity. Later in the study the minimal epitopes defined using >40% purity peptides were confirmed with >90% purity peptides, which were used for refolding soluble SLA-I.

### Manufacture of soluble pSLA-I for tetramers

The minimal epitopes as described above were refolded with the Babraham SLA-I alleles. SLA-1*14:02, SLA-2*11:04 and porcine β2M constructs were synthesized with 5’ EcoR1 and 3’ BamH1 restriction sites (see below) and cloned into vector pUC57-Amp (Genewiz LLC, U.S.) and inserted into pGMT7 bacterial expression vectors, under the control of the T7 RNA polymerase promoter. These vectors were then transformed into One Shot (TOP10): *E*. *coli* (Invitrogen) competent cells and grown on carbenicillin treated agar plates. Plasmid DNA was isolated from individual bacterial colonies and its integrity was confirmed by sequencing, using T7 primers (Eurofins Genomics). Sequence-confirmed plasmids were transformed into Rosetta 2(DE3) pLysS: *E*. *coli* (Novagen, Merck Millipore) competent cells and inclusion bodies were produced as previously described [34]. Cells were induced with IPTG (Fisher Scientific, U.S.) once OD reached 0.5 and protein concentration was measured on a spectrophotometer prior to refolding. SLA-1*14:02 or SLA-2*11:04 (both with a biotin tag), porcine or human β2M and NP peptides (>90% purity, Peptide Protein Research Limited) were refolded and purified as previously described for human HLA-I [34]. Each refold was incubated at 4°C for at least 6 h (usually overnight) before dialysis in 10 mM TRIS pH 8.1. The protein was gel-filtrated into PBS. All purification steps were performed on an AKTA FPLC machine using the Unicorn software (GE Healthcare). pSLA complexes were biotinylated prior to gel filtration with the BirA biotin-protein ligase standard reaction kit as per the manufacturer’s instructions (BirA500, Avidity LLC.).

SLA-1*14:02 (Biotinylation site):

MGPHSLSYFSTAVSRPDRGDSRFINFLUENZAGYVDDTQFVRFDSDAPNPRMEPRAPWIQQEGQEYWDRNTRNVMGSAQINRVNLKTLRGYYNQSEAGSHTLQWMYGCYLGPDGLLLRGYDQFAYDGADYLALNEDLRSWTAADMAAQISKRKWEAADAAEHWRSYLQGTCVESLRRYLQMGKDTLQRAEPPKTHVTRHPSSDLGVTLRCWALGFHPKEISLTWQREGQDQSQDMELVETRPSGDGTFQKWAALVVPPGEEQSYTCHVQHEGLQEPLTLRWDPGLNDIFEAQKIEWHE

SLA-2*11:04 (Biotinylation site):

MGPHSLSYFYTAVSRPDRGEPRFINFLUENZAGYVDDTQFVRFDSDAPNPRMEPRAPWIQQEGQDYWDRETQIQRDNAQTFRVNLRTALGYYNQSEAGSHTFQSMYGCYLGPDGLLLRGYSQYGYDSADYIALNEDLRSWTAADTAAQITKRKWEAADEAEQWRSYLQGLCVEGLRRYLEMGKDTLQRAEPPKTHVTRHPSSDLGVTLRCWALGFYPKEISLTWQREGQDQSQDMELVETRPSGDGTFQKWAALVVPPGEEQSYTCHVQHEGLQEPLTLRWDPGLNDIFEAQKIEWHE

Porcine β2M:

MVARPPKVQVYSRHPAENGKPNYLNCYVSGFHPPQIEIDLLKNGEKMNAEQSDLSFSKDWSFYLLVHTEFTPNAVDQYSCRVKHVTLDKPKIVKWDRDH

### Tetramer staining of T-cells from Babraham pigs 625 and 650

#### Tetramer assembly

Soluble biotinylated NP peptide SLA-I were assembled into tetramers with streptavidin-PE (Life Technologies) as previously described [35] followed by the addition of protease inhibitors (1:100 dilution of set 1; Merck) and PBS to give a final concentration of 0.1 μg/μL (with respect to the pSLA-1 component). Tetramers were stored and used as previously described [36].

#### Tetramer staining clones

T-cells clones from Babraham pigs 625 (Bab.625 and KT22.625) and 650 (KT7.650 and KLT.650) were treated with or without 50 nM protein kinase inhibitor (PKI) (Dasatinib, Axon Medchem) as previously described [37], directly followed by staining with pSLA-I tetramer (0.3 μg per sample with respect to the pSLA-I component), on ice for 30 min. PKI was stored at -80°C in 1 mM DMSO one-use aliquots and dilutions were made freshly in PBS for each experiment. The cells were washed with PBS and stained in approximately 50 μL of residual PBS with LIVE/DEAD Violet stain Vivid (1:40 dilution in PBS then 2 μL per sample) (Life Technologies) for 5 min at RT, followed by mouse anti-pig CD8β-FITC (PPT23, Bio-Rad) for 20 min on ice. T-cell clones were gated for size (lymphocyte gate: forward scatter height versus side scatter area), single (forward scatter height versus forward scatter area) and CD8β^+^ Vivid^neg^ cells then displayed as histograms of tetramer fluorescence.

#### Ex vivo staining

PBMCs, BAL and TBLN cells were stained with pSLA-I tetramer as above for clones. An optimized tetramer staining protocol was used; PKI as above for clones and 10 μg/mL of a mouse anti-PE antibody (PE001; Biolegend) added post tetramer staining for 20 min on ice [38]. Cells were washed in PBS and stained with Vivid as above. Surface marker Abs were added to residual buffer for 20 min on ice; 1.5 μL mouse anti-pig CD3ε PE-Cy7 (BB23-8E6-8C8; BD Biosciences); 3 μL mouse anti-pig CD4 AF647 (74-12-4; BD Biosciences); 1.5 μL mouse anti-pig CD8β FITC; and 2 μL mouse anti-human CD14 Pacific Blue (PB) (TUK4; Bio-Rad). The Biorad TUK4 anti-human CD14 clone cross-reacts with pig CD14. Where required, cells were fixed at this stage with 2% paraformaldehyde (PFA) on ice for 20 min. The sequential gating strategy used for pSLA-I tetramer analysis of tissue samples was based on those used for studying T-cell responses in humans and adapted depending on the availability of conjugated pig antibodies; Gate 1: lymphocytes; Gate 2: single cells; Gate 3: viable T-cells (CD3^+^) distinguished from monocytes (CD14^+^) and dead cells, thereby avoiding cells that may bind tetramers non-specifically; and Gate 4: selecting cells based on staining with anti-CD8β and anti-CD4 antibodies, therefore excluding CD8^neg^ and CD4^neg^ cells, such as B-cells from analysis, as they may also bind tetramers non-specifically (**S1 Fig**). The CD4+CD8β- cells (which would include the MHCII-restricted CD4+ CD8αα+ cells) then acted as an internal control T-cell subset to assess the extent of background tetramer staining relative to the CD8β subset.

### Tetramer staining of T-cells from Babraham pigs 1, 2, 6, 7 and 8

#### Vaccination

Four Babraham pigs were divided into two groups, pigs 1 and 2 were left unvaccinated and pigs 6, 7 and 8 received H1N1-S-FLU by aerosol (~ 2 x 10^7^ TCID_50_ per dose) using a InnoSpire Deluxe Philips Respironics nebulizer fixed to a small-sized anaesthetic mask held over the animal’s nose and mouth. Vaccinated pigs received an H1N1-S-FLU boost at day 28. All pigs were euthanized at day 57 (day 28 post boost) and BAL harvested and cryopreserved as above.

#### Ex vivo tetramer staining

BAL cells from pigs 1, 2, 6, 7 and 8 were stained as described above for pigs 625 and 650.

### Functional and tetramer analysis of T-cells from Babraham pigs 741, 742, 744 and 745

#### Infection

Babraham pigs 742, 744 and 745 were experimentally infected with live isolate of pandemic H1N1 swine virus [A/Swine/Eng/1353/2009] intranasally at 1.5x10^7^ PFU per dose and a fourth control pig (741) was left uninfected. Control pig 741 was euthanized at day 0 and the infected pigs at either day 5 (744) or day 14 post infection (742 and 745), with BAL derived cells harvested for cryopreservation as above.

#### Ex vivo ELISPOT assay

Responses to the NP epitopes identified using pigs 625 and 650 were tested *ex vivo* for pig 745 as exogenous peptide. ELISPOT plates (MSIPS4510, Merck Millipore) were coated with 5 μg/mL mouse anti-pig IFNγ Ab (clone P2G10, BD Biosciences) and incubated at 37°C for 4 h. Plates were washed with PBS and blocked at RT for 1 h with R10 medium. BAL samples were defrosted as previously described and cultured in R5 medium (200,000 cells/well) with 15,000 of the Babraham kidney epithelial cell line (details above) to act as antigen presenting cells. NP peptides were used at 10^-5^ M, H1N1-S-FLU was used at 3.5 x10^6^ TCID_50_ and live MDCK cell-grown A/Sw/Eng/1353/09 used at titre 6 x 10^7^ pfu/mL. Where possible, all conditions were performed in duplicate. The plates were incubated at 37°C for 16–18 h, washed and then incubated with sterile H_2_O at RT for 10 min before further washing. Plates were then incubated with 1 μg/mL biotin mouse anti-pig IFNγ Ab (clone P2C11, BD Biosciences) at RT for 2 h and then washed. Plates were incubated with 50 μL (1:1000) Streptavidin-Alkaline phosphatase (BioRad) per well at RT for 2 h. Plates were then developed according to the manufacturer’s instructions (AP conjugate substrate kit, BioRad). An Immunospot analyzer (Cellular Technology Limited, U.S.) was used to count the number of spot forming cells (SFC) per well, which were scaled (X5) to 10^6^ cells based on the number of cells added per well.

#### Ex vivo tetramer staining

BAL cells from pigs 741, 742 and 744 were stained as described above for pigs 625 and 650.

### Babraham SLA-I peptide anchor residues and motifs

#### Crystal structures

Constructs were designed and synthesized as described above but without the biotin tag sequence (shown underlined above). Refolding of soluble pSLA-I is also described above, but with gel filtration in to crystal buffer used for the pSLA-I, rather than PBS. The NP pSLA-I (with either human or porcine β2M) were subsequently concentrated to ~10 mg/mL in crystal buffer. Crystallisation screens were set up using a Gryphon crystallography robot (Art Robbins Instruments) via the sitting drop technique in 96-well Intelli-plates (Art Robbins Instruments). 60 μL of each screen condition was dispensed in the deep well followed by a 1:1 ratio of screen:protein volume in the sitting drop. Protein crystals were grown by vapour diffusion at 18°C and visualized using RockImager and RockMaker software (Formalatrix). The following crystallisation screens, consisting of 96 different buffer compositions, were set up for each protein; JBScreen Basic HTS (Jena Bioscience), PACT premier HT-96 (Molecular Dimensions) and TCR optimized protein screen (TOPS) developed at Cardiff University [39]. Crystals were harvested and placed into liquid nitrogen and transported to the Diamond Light Source (Oxfordshire, U.K.) for X-ray diffraction data collection. Beamlines I02, I03, I04 and I04-1 were used for data collection. Reflection intensities were estimated with XDS [40] as implemented in the XIA2 package [41] and the data were scaled, reduced and analyzed with AIMLESS and TRUNCATE in the CCP4 package [42,43]. All structures were solved by molecular replacement with PHASER [44] using 3QQ3 as a starting model [45]. Sequences and models were adjusted with COOT [46] and the models refined with REFMAC5 [47]. Graphical representations were prepared in PYMOL (The PyMOL Molecular Graphics System, Version 1.8 Schrödinger, LLC.). The five X-ray structures solved in this study were deposited into the Protein Data Bank (http://www.rcsb.org/pdb/). The models SLA-1-EFEDLTFLA-pβ2M, SLA-1-DFEREGYSL-pβ2M, SLA-2-IAYERMCNI-pβ2M, SLA-1-EFEDLTFLA-hβ2M and SLA-1-DFEREGYSL-hβ2M were assigned accession codes 5NPZ, 5NQ0, 5NQ2, 5NQ3 and 5NQ1 respectively.

#### T-cell assays using peptide anchor variants

SLA-1*14:02 and SLA-2*11:04 restricted clones grown from pigs 625 (clones: KT22.625 and Bab.625) and 650 (clones: KT4.650 and KLT.650) were used to test their respective NP peptides that had been substituted at the P2 or P9 peptide anchor residues with each of the proteogenic amino acids. Decreasing concentrations of each peptide were tested in titrations assays with T-cell activation assessed by MIP-1β ELISAs as described above.

### Epitope prediction using Babraham pigs 742 and 745

Predicted epitopes were obtained by scanning five PR8 influenza proteins; Matrix 1 and 2, NP, and polymerase basic proteins 1 and 2; for 9 amino acid length peptides that fit the anchor motifs we defined for SLA-1*14:02 and SLA-2*11:04.

#### Ex vivo and cultured ELISPOT assays

Babraham pigs 742 and 745 infected with live influenza virus as described above were used to test for T-cell responses towards the predicted epitopes using ELISPOTs as described above. BAL cells from pig 745 were defrosted and 200,000 cells per well incubated with 10^-5^M peptide, virus or alone. In a separate experiment, BAL cells from pig 742 was cultured with predicted epitopes identified from pig 745 to create T-cell lines, using the same method as described above for pigs 625 and 650 but without CD8β sorting. After 14 d the T-cell lines were washed with R0 and divided equally between the necessary conditions for an ELISPOT but without counting the cells from each condition, as cell numbers were low. SFCs were scaled (X5) to 1x10^6^ cells for pig 745, and the actual number of SFCs displayed for pig 742.

## Results

### Determination of epitopes recognized by CD8 T-cells following influenza vaccination

**Fig 2:**
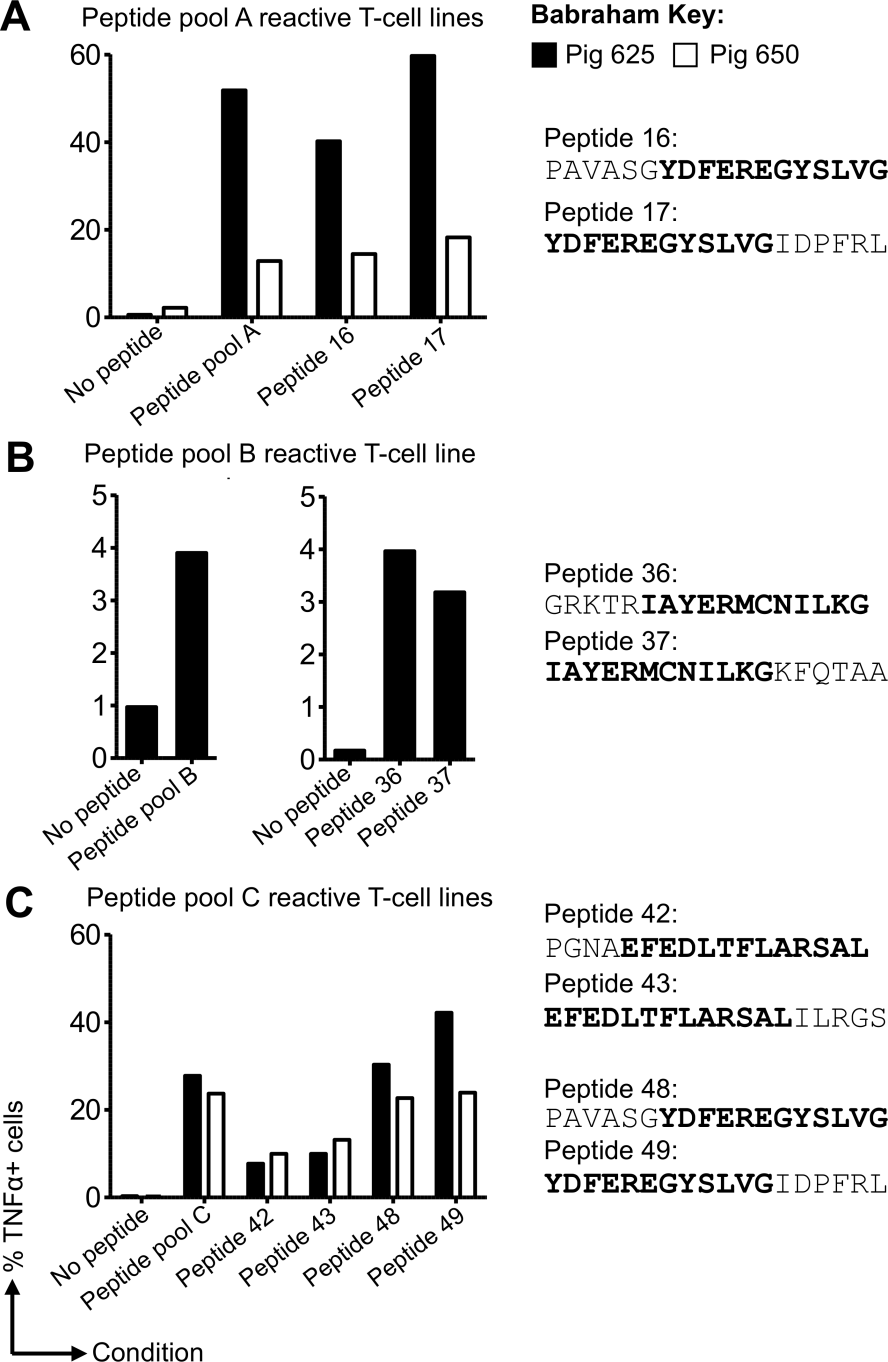
Generation of influenza-specific CD8β T-cell lines from Babraham pigs simultaneously immunized with H5N1-S-FLU and Sp/Sw H1N1. CD8β T-cells purified from the peripheral blood mononuclear cells of Babraham pigs 625 (filled bars) and 650 (open bars) were stimulated *in vitro* with pools (four pools: A, B, C and D) of overlapping peptides (81 in total, **S2 Table**) from the NP of S-FLU (PR8). Two weeks post stimulation the cells were tested for reactivity towards individual peptides from each pool. Intracellular cytokine staining for TNF was performed following a 5 h incuabtion with no peptide (DMSO control) or peptides (2 μM). All associated flow cytometry data is shown in **S2 Fig** (pig 625) and **S3 Fig** (pig 650) with the percentage of TNF producing cells displayed here. (**A**) T-cell lines generated against pool A mapped to individual peptides 16 and 17 (sequences shown with overlap region in bold). (**B**) Reactivity for pool B for pig 625 (left graph) had been seen in previous experiments with indications for reactivity towards peptides 36 and 37, thus lines were successfully generated against individual peptides 36 and 37 (right graph). (**C**) Lines responsive to pool C mapped to individual peptide 42, 43, 48 and 49. T-cell clones were grown directly from these lines by limiting dilution or first by enriching for peptide specific T-cells using a TAPI-0 assay (**S1 Fig**) and flow cytometry.

Studies in mice and humans have demonstrated that T-cells specific for conserved internal NP are associated with protective heterotypic influenza immunity [7–9,48]. Therefore, we initially set out to define T-cell epitopes from NP in pigs simultaneously immunized with H5N1 S-FLU intranasally and inactivated H1N1 [A/Swine/Spain/SF11131/2007] (Sp/Sw) strain intramuscularly (Babraham pigs 625 and 650) (**Fig 1**). Overlapping peptides from the NP of S-FLU (PR8), listed in **S4 Table**, were used to stimulate CD8β T-cells and peptide-specific T-cell responses identified by intracellular cytokine staining for TNF. The purity of CD8β T-cells prior to culture was typically >85% with similar proportions of CD8β seen after two weeks of culture (**S2 and S3 Figs**). T-cell lines were grown to peptide pools A, B and C for Babraham pig 625 (**S2 Fig**) and peptide pools A and C for Babraham pig 650 (**S3 Fig**). Background activation was minimal for the no peptide controls in both pigs, ranging between 0.17% and 2.24%. The T-cell line data are summarized in **Fig 2**and show substantial TNF production in response to peptide pools A (51.8% and 12.9%, respectively in pig 625 and 650) and pool C (27.8% and 23.7% respectively in pig 625 and 650). Individual overlapping peptides from these pools were used in activation assays to identify the immunogenic peptide(s). For both pigs 625 and 650 the responses to peptide pool A mapped to neighbouring peptides 16 and 17 (**Fig 2A**), which share an ‘overlap’ peptide region, and similarly for pool C to neighbouring peptides 42/43 and 48/49 (**Fig 2C**). The high percentage of T-cell reactivity to peptide pools A and C enabled direct procurement of T-cell clones by limiting dilution. For pool B, peptide-responsive T-cells from pig 625 were sorted based on TNF expression (TAPI-0 assay) by flow cytometry prior to cloning (**S1 Fig**). The TAPI-0 assay detected cell surface bound TNF for both mitogen and peptide stimulated cells (**S1 Fig**) and unlike ICS has the advantage of allowing live cells to be sorted post antigenic stimulation. The following CD8β T-cell clones were grown from the T-cell lines (listed in **Fig 1**): clones 4 and 7 from pig 650 (named KT4.650 and KT7.650) both recognizing peptides 48 and 49; clones S and T also from pig 650 (named KTS.650 and KLT.650) both recognizing peptides 42 and 43; clones 22 and 13 from pig 625 or 650 respectively (named KT22.625 and KT13.650) both recognizing peptides 16 and 17; and clones ‘Sue’ and ‘Bab’ from pig 625 both recognizing peptides 36 and 37.

**Fig 3:**
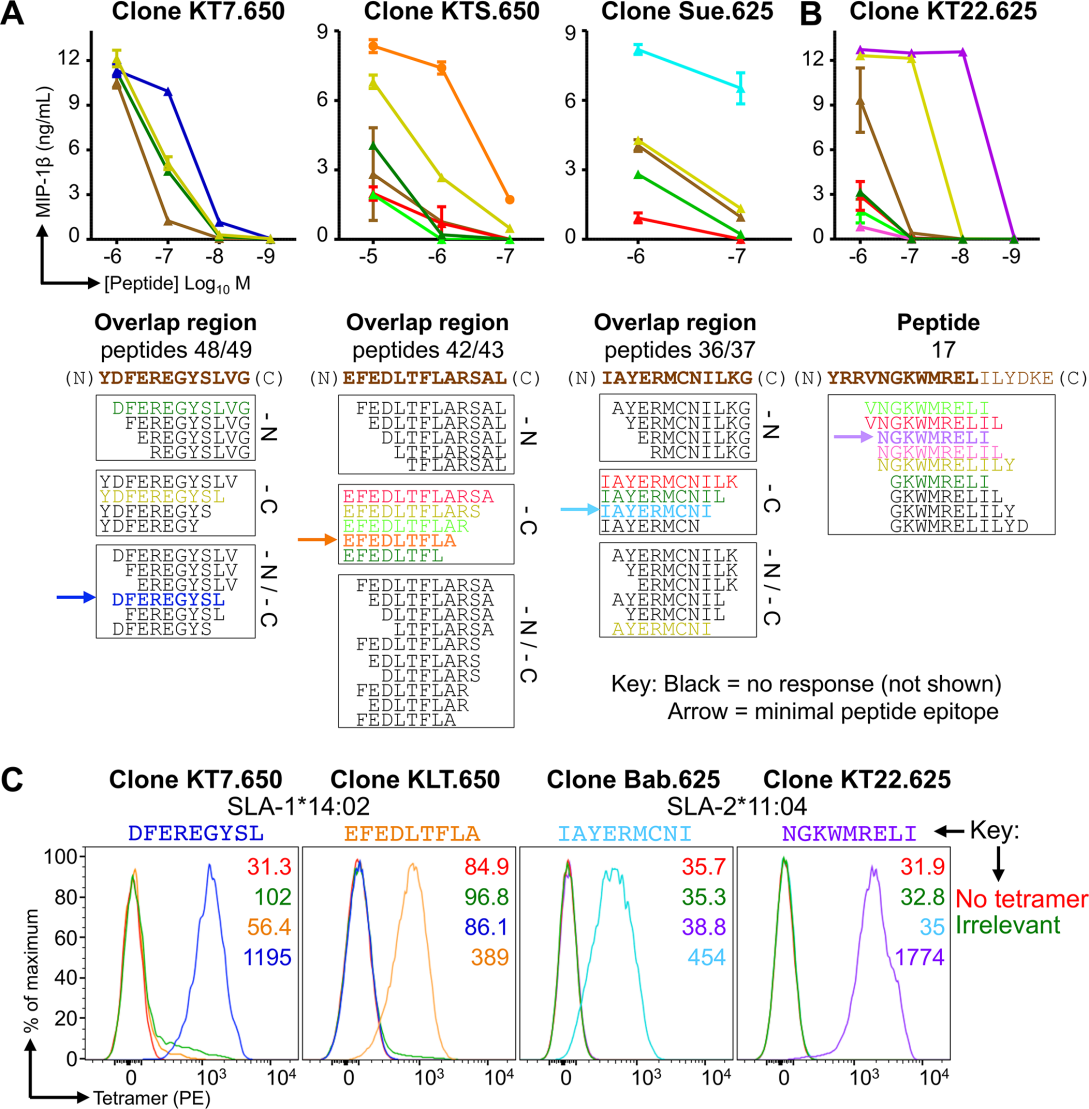
Minimal epitope identification and pSLA tetramer staining of influenza-specific T-cell clones. CD8β clones were grown from T-cell lines generated from Babraham pigs 625 and 650 using overlapping peptides from the NP of S-FLU (PR8) (**Fig 2**). The .650 or .625 indicates the pig the clones were grown from. (**A**) Clones KT7.650, KTS.650 and Sue.625 responded to the overlapping peptide regions shown in brown text below each graph. Clones were incubated with decreasing concentrations (upper panel) of truncated versions of the respective peptide (sequences shown in the lower panel). Truncations were performed at the amino (N) and/or carboxyl (C) terminal ends of each peptide as indicated (lower panel). MIP-1β ELISAs were performed to assess T-cell activation after overnight incubation. Peptides that did not elicit a repsonse are shown in black and are not displayed on the graphs. The minimal peptide epitope is indicated by the arrow. (**B**) Using a similar approach as in (A). In order to induce maximal activation and define the minimal epitope, clone KT22.625 (right) required the isoleucine from peptide 17, which was not present in the overlap region of 16/17. (**C**) The minimal epitopes defined in (A and B) were refolded with both SLA-1*14:02 and SLA-2*11:04, with successful refolding determining restriction. pSLA-I tetramers were assembled and used to stain clones with respective peptide specificity, as shown. Irrelevant tetramers: SLA-1*14:02-AFAAAAAAL and SLA-2*11:04-AGAAAAAAI.

The overlapping region shared by neighbouring peptides, such as **YDFEREGYSLVG** from peptides 48 and 49 **(**PAVASG**YDFEREGYSLVG** and **YDFEREGYSLVG**IDPFRL respectively, overlap in bold, **Fig 2**) were used to map the minimal epitope recognized by each T-cell clone (**Fig 3**). Although human and mouse MHC class I molecules can present peptides of 8–14 amino acids in length, most (>70%) of CD8+ T-cell epitopes are 9 amino acids in length with ~20% being 10 amino acids in length [49]. Similarly, the limited studies in pigs to date have identified responses to peptides of 9 or 10 amino acid in length [22,45,50–52]. We truncated the shared region from overlapping peptides identified in **Fig 2**to a length of 8 amino acids to establish the minimal epitopes (**Fig 3**). Titration of truncated peptides in T-cell assays with three different clones indicated that they responded best to the 9 amino acid long sequences: DFEREGYSL (from 48 49), EFEDLTFLA (42 43) and IAYERMCNI (36 37) (**Fig 3A**). A fourth nonamer epitope, NGKWMRELI, required the C-terminus Ile from peptide 17, which was not present in the overlap region between peptides 16 and 17, in order to induce maximum T-cell activation (**Fig 3B**). Other clones specific for these peptides gave similar results. To determine which of the two Babraham SLA molecules presented these epitopes we refolded each with β2M and the extracellular domain of either SLA-1*14:02 or SLA-2*11:04. Each SLA heavy chain refolded with two of the four peptides so we can be confident that DFEREGYSL and EFEDLTFLA are restricted by SLA-1*14:02 while IAYERMCNI and NGKWMRELI are restricted by SLA-2*11:04. We also refolded each of the Babraham SLA-I with irrelevant peptides (sequences in figure legends) to use as control tetramers, which allowed the extent of background tetramer staining to be assessed and also aided in the placement of influenza tetramer^+^ gates during analysis (**S1 Fig**). The refolded monomers were assembled into tetramers to confirm their ability to stain respective T-cell clones. Influenza-specific clones clearly stained with pSLA-I tetramers (**Fig 3C**) confirming the peptide recognized, the restricting SLA molecule and successful production of pSLA-I tetramers.

### pSLA-I tetramers reveal influenza specific CD8 T-cells in *ex vivo* samples from S-FLU vaccinated Babraham pigs

As we were interested in staining cytotoxic T-cells (CD8β^+^) with pSLA-I tetramers, we firstly tested available anti-pig CD8β Ab clones (PG164A and PPT23 [53,54]) on Babraham PBMCs. The Abs detected similar proportions of CD8β cells with distinguishable CD8β^+^/CD8α^+^ cells from CD8β^neg^/CD8α^+^ cells (**S4 Fig**). This discrimination is important given the expression of CD8α by many cell subsets in swine. Ab Clone PPT23 was used for *ex vivo* staining, as it was commercially available conjugated to FITC and therefore compatible with PE tetramers. PE is our favored fluorochrome for tetramer staining due to its relative brightness [55]. As part of the gating strategy for pSLA-I tetramer analysis we included CD4 cells to act as an irrelevant T-cell subset (**S1 Fig**) and as a result we observed that a small proportion of the CD4 cells co-stained for CD8β (Range: 0.04–1.25%. Mean: BAL 0.27% n = 10, PBMC 0.55% n = 3, TBLN 0.87% n = 2) (**S3 Table**). Initial testing of pSLA-I tetramer staining showed that inclusion of protein kinase inhibitor [37] and anti-fluorochrome antibody [38] improved the detection of pig T-cells with pSLA-I tetramers (**S4 Fig**), therefore these conditions were used for all subsequent tetramer staining.

**Fig 4:**
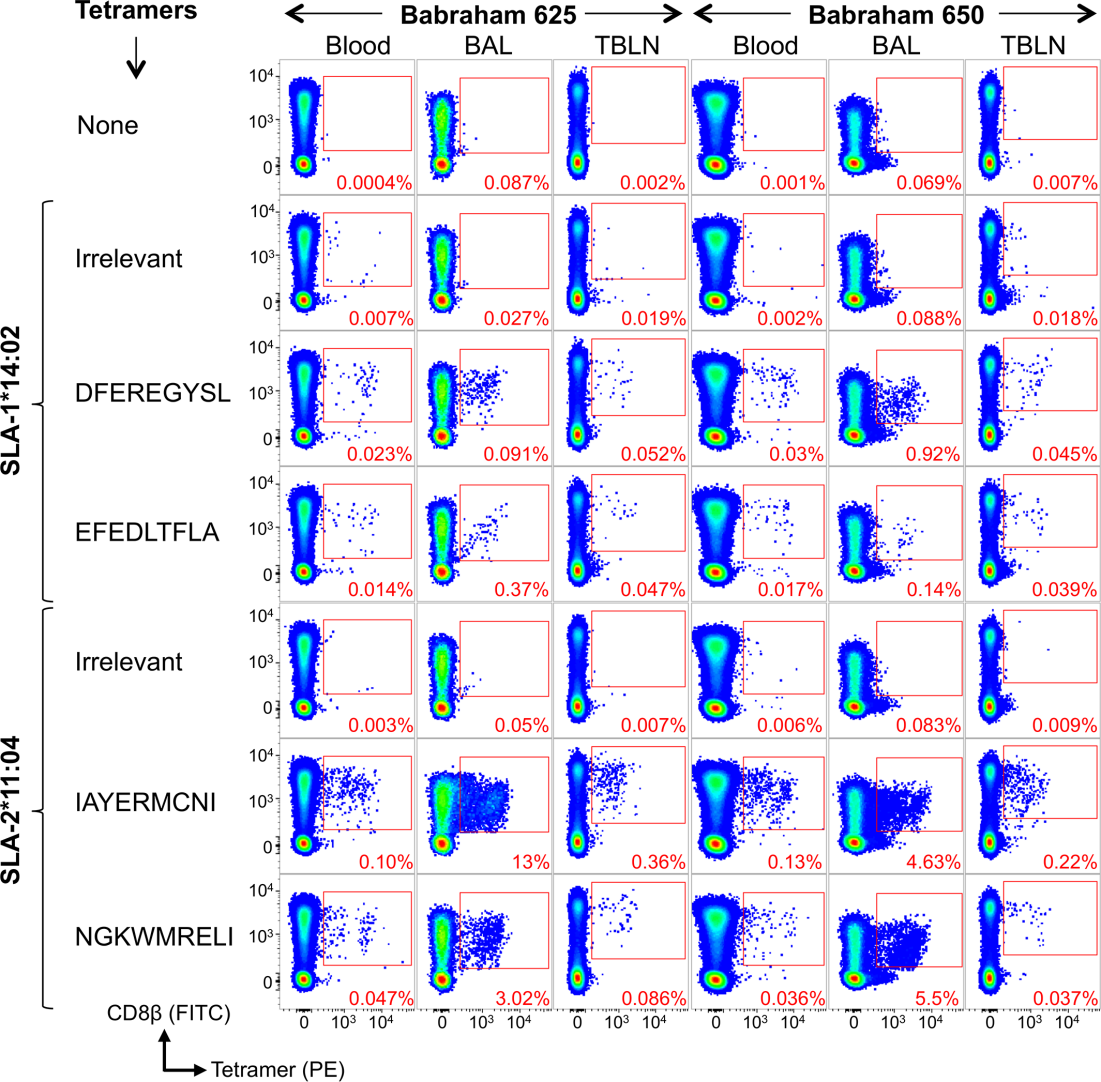
Nuceloprotein pSLA-I tetramer staining of tissues from influenza vaccinated Babraham Pigs. Babraham pig 625 (left panel of 21 plots) and 650 (right panel of 21 plots) received H5N1 S-FLU intranasally and inactivated H1N1 virus [A/Swine/Spain/SF11131/2007] with montanide adjuvant intramuscularly, followed by a boost at day 25 using the same preparation. Pigs were culled at day 38 (day 13 post boost) and blood, bronchoalveolar lavage (BAL) and tracheobronchial lymph nodes (TBLNs) harvested and frozen as single cell suspensions. Tetramer staining was performed on thawed cells from the blood, BAL and TBLN using a no tetramer control, and staining with Irrelevant and nucleoprotein peptide tetramers. The sequences for the nucleoprotein peptides are shown. Irrelevant tetramers: SLA-1*14:02-AFAAAAAAL, SLA-2*11:04-AGAAAAAAI (pig 625) and SLA-2*11:04-GAGGGGGGI (pig 650). Gating strategy: lymphocytes, single cells, viability (Vivid^neg^) CD3^+^ CD14^neg^ then CD8β^+^ CD4^+^ and displayed as CD8β versus tetramer (**S1 Fig**).

Tetramers refolded with four NP peptides were used to stain *ex vivo* samples from Babraham pigs 625 and 650, which had been vaccinated with H5N1 S-FLU and inactivated H1N1 [A/Swine/Spain/SF11131/2007] (Sp/Sw). The T-cell clones used to define the NP epitopes in this study were grown from Babraham pigs 625 and 650. Cryopreserved PBMCs, and cells derived from the BAL and TBLN (day 13 post boost) were stained with irrelevant and four influenza pSLA-I tetramers. Antigen-specific CD8β T-cells were detectable in both pigs and in all samples (**Fig 4**). In pig 625, SLA-1*14:02 DFEREGYSL and EFEDLTFLA responses accounted for 0.023% and 0.014% in PBMCs, 0.091% and 0.37% in the BAL and 0.052% and 0.047% in the TBLN of CD8β T-cells, respectively. A higher proportion of DFEREGYSL tetramer^+^ cells (0.92%) were seen in the BAL of pig 650. Responses to SLA-2*11:04 restricted epitopes IAYERMCNI and NGKWMRELI were higher in magnitude in both pigs. In PBMCs, IAYERMCNI accounted for 0.1% and 0.13% of CD8β T-cells in pigs 625 and 650 respectively, and NGKWMRELI accounted for 0.047% and 0.036%. The largest responses were detected in the BAL with IAYERMCNI binding 13% and 4.63% of CD8β T-cells in pigs 625 and 650, respectively. Overall, these results indicate that T-cell clones isolated from PBMCs are representative of T-cells distributed in lymphoid and non-lymphoid tissues.

### S-FLU and live swine influenza elicit CD8β T-cells responses in Babraham pigs that were detectable with pSLA-I tetramers

**Fig 5:**
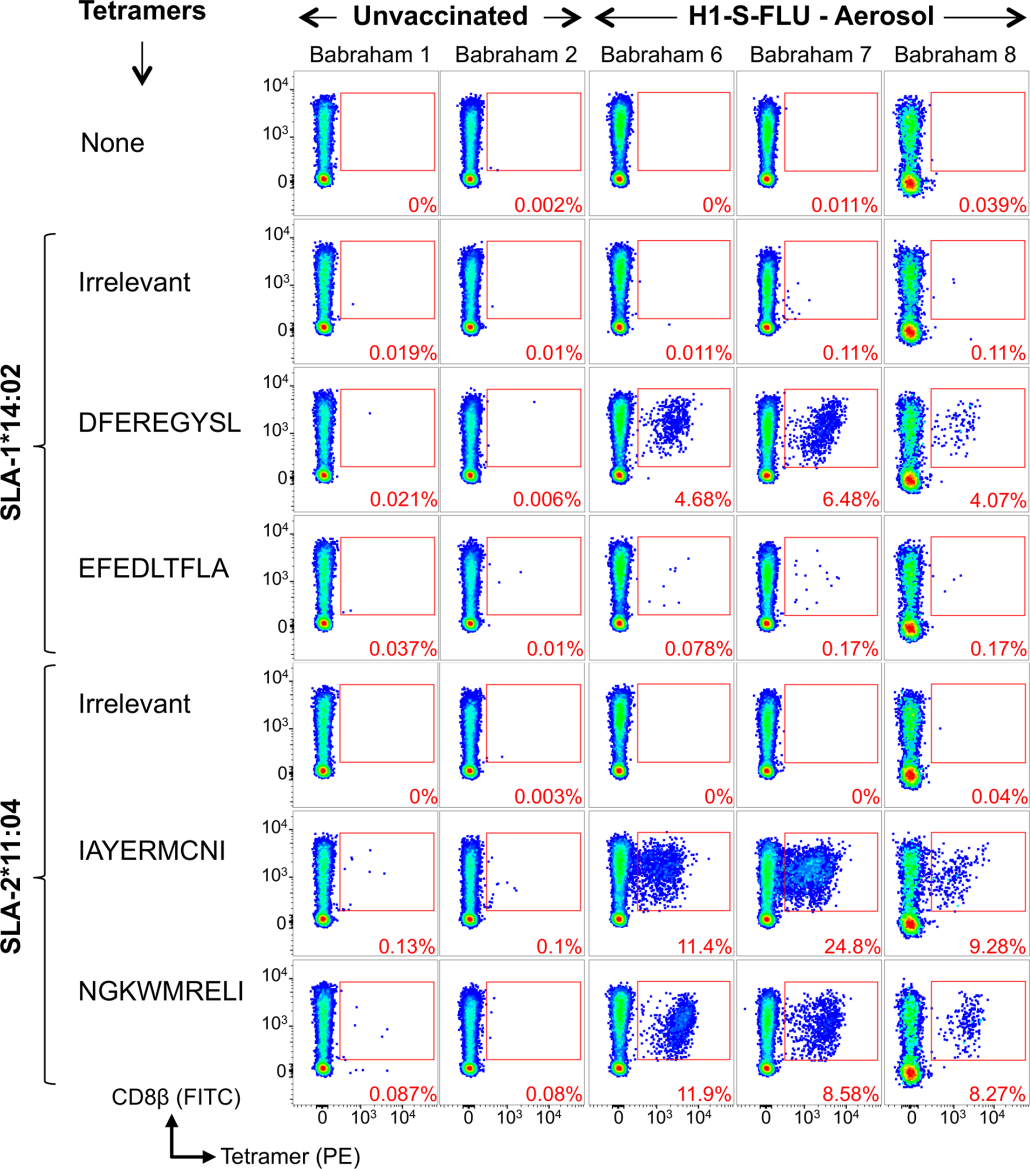
Nuceloprotein pSLA-I tetramer staining of bronchoalveolar lavage samples from Babraham pigs vaccinated with H1N1 S-FLU. Babraham pigs were either left unvaccinated (1 and 2) or received H1N1 S-FLU via aerosol administration (6, 7, 8). H1N1 S-FLU vaccinated animals received a boost at day 28 with the same vaccine. Animals were culled and bronchoalveolar lavage harvested at day 57. Nucleoprotein and irrelevant peptide SLA-I tetramer staining was performed on thawed bronchoalveolar lavage samples and the percentage of tetramer^+^ cells of CD8β^+^ cells displayed in red. The sequences of the nucleoprotein peptides are shown. Irrelevant tetramers: SLA-1*14:02-AFAAAAAAL and SLA-2*11:04-GAGGGGGGI. Gating strategy: lymphocytes, single cells, viability (Vivid^neg^) CD3^+^ CD14^neg^ then CD8β^+^ CD4^+^ and displayed as CD8β versus tetramer (**S1 Fig**).

**Fig 6:**
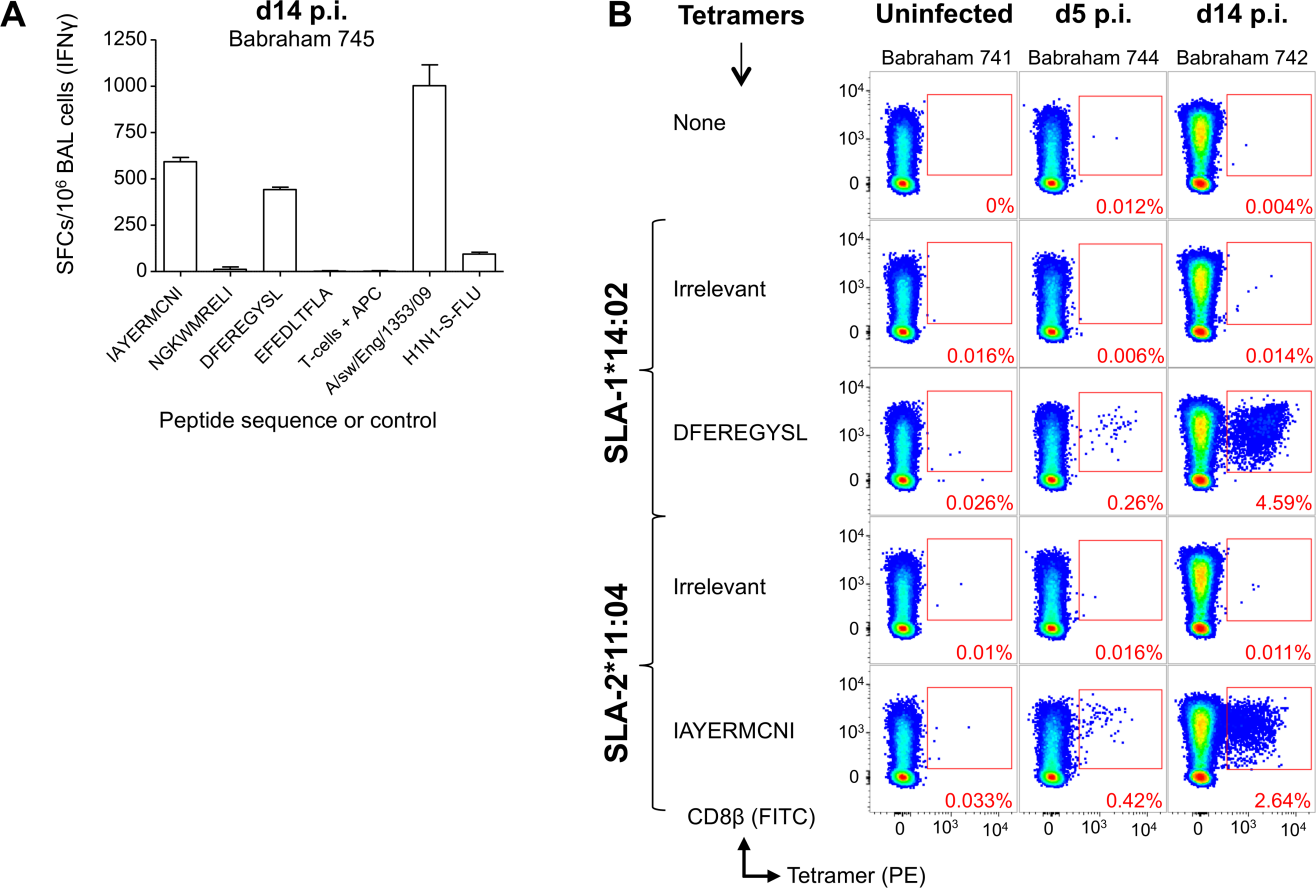
Identification of influenza-specific T-cells in the bronchoalveolar lavage from Babraham pigs infected with pandemic H1N1 swine influenza virus. Babraham pigs were either left uninfected (741) or infected intranasally with pandemic H1N1 [A/sw/Eng/1353/09] (742, 744, 745). The pigs were culled on day 0 (741), 5 (744) or 14 (742 and 745) post infection. (**A**) 200,000 bronchoalveolar lavage cells from pig 745 (infected, day 14 cull) were incubated alone, with 10^-5^M peptide, or virus for 16–18 h. A Babraham kidney cell line was included in each well (15,000 per well) to act as antigen presenting cells. All conditions were performed in duplicate and spot forming cells (SFCs) detected by IFNγ ELISPOT and displayed as mean +SEM and scaled (X5) to 10^6^ BAL cells. (**B**) Irrelevant and nucleoprotein peptide-SLA-1*14:02 and SLA-2*11:04 tetramer staining was performed on thawed bronchoalveolar lavage samples and the percentage of tetramer^+^ cells of CD8β cells displayed in red. Nucleoprotein peptide sequences are shown. Irrelevant tetramers were SLA-1*14:02-AFAAAAAAL and SLA-2*11:04-AGAAAAAAI. Gating strategy: lymphocytes, single cells, viability (Vivid^neg^) CD3^+^ CD14^neg^ then CD8β^+^ CD4^+^ and displayed as CD8β versus tetramer (**S1 Fig**).

As the T-cell clones used to define the NP epitopes were derived from Babraham pigs 625 and 650 immunized simultaneously by parenteral and mucosal routes with two different vaccines, we next sought to test whether T-cells with the same specificities could be detected in pigs vaccinated with S-FLU by a single delivery route. We vaccinated 3 Babraham pigs (6, 7 and 8) with H1N1 S-FLU by aerosol, which we have previously shown to significantly reduce the viral load in nasal swabs and lungs in outbred pigs following challenge with a swine isolate of pdmH1N1 virus [27]. The pigs were vaccinated twice, 4 weeks apart and euthanized 28 days later and the BAL harvested. Babraham pigs 1 and 2 were left unvaccinated. BAL cells were stained with tetramers (**Fig 5**) and very large CD8β^+^/tetramer^+^ populations were seen in the H1N1 S-FLU vaccinated animals for three epitopes, DFEREGYSL, IAYERMCNI and NGKWMRELI. In pig 7, almost 40% of CD8β T-cells in the BAL responded to just these three NP epitopes, indicating the efficiency of aerosol delivery at generating local long-lived immune responses. In contrast, the control pigs showed no substantial responses above background to the influenza epitopes (**Fig 5**for pigs 1 and 2). A relatively smaller response was observed to EFEDLTFLA in the BAL through SLA-1*14:02 in the vaccinated pigs. The presence of antigen specific T-cells within the BAL of the vaccinated pigs (n = 3) did not alter the proportion of CD3 (7.19%) or CD8β (1.62%) cells of total cells when compared to the control pigs (n = 2: CD3 9.44% and CD8β 1.37%), with the other vaccinated/infected Babraham pigs (n = 5) of this study having similar levels of CD3 (8.62%) and CD8β (1.28%) cells in the BAL (**S3 Table**). We next investigated whether responses to these epitopes could be detected following swine influenza infection. Three Babraham pigs were infected intranasally (742, 744, 745) with a swine isolate of pdmH1N1 [A/Swine/Eng/1353/2009] and one pig left uninfected (741). BAL samples were taken from infected pigs upon culling at days 5 and 14 post infection. Due to limited sample availability, the four NP epitopes were first tested by IFNγ ELISPOT *ex vivo* on BAL cells isolated from the day 14 sample from pig 745 (**Fig 6A**). This identified strong responses to two of the four epitopes, IAYERMCNI and DFEREGYSL, inducing on average 592 and 442 SFC per 10^6^ BAL cells, respectively. These two epitope sequences are conserved (DFEREGYSL is identical and IAYERMCNI has a conservative single residue change from I to V at P1 (**S2 Table**)) between H5N1 or H1N1 S-FLU and the swine influenza strain used in this infection. The other epitopes identified in S-FLU vaccinated pigs, NGKWMRELI and EFEDLTFLA, are not as conserved in the swine influenza virus used. This difference in sequence is likely to account for the lack of responses observed especially for the EFEDLTFLA epitope, which has the sequence E**I**EDL**I**FLA in the virus [A/Swine/Eng/1353/2009] and is not predicted to bind to the restricting SLA molecules (see below and **S2 Table**). Peptide-SLA-I tetramer staining was then performed and demonstrated large responses to epitopes IAYERMCNI and DFEREGYSL at 14 days post infection, with 2.64% and 4.59% of CD8β T-cells staining positive for each of these epitopes, respectively (**Fig 6B**). These T-cell responses were smaller but readily detectable at 5 days post infection and were negligible in the unvaccinated pig. These results indicate that the pSLA-I tetramers can detect a high proportion of CD8β T-cells specific for conserved NP epitopes in the BAL of pigs following vaccination or infection and illustrate how antigen specific T-cells are concentrated at sites of infection or sites of mucosal immunization.

### Structural analyses of pSLA-I complexes reveal anchor residues for SLA-1*14:02 and SLA-2*11:04

**Fig 7:**
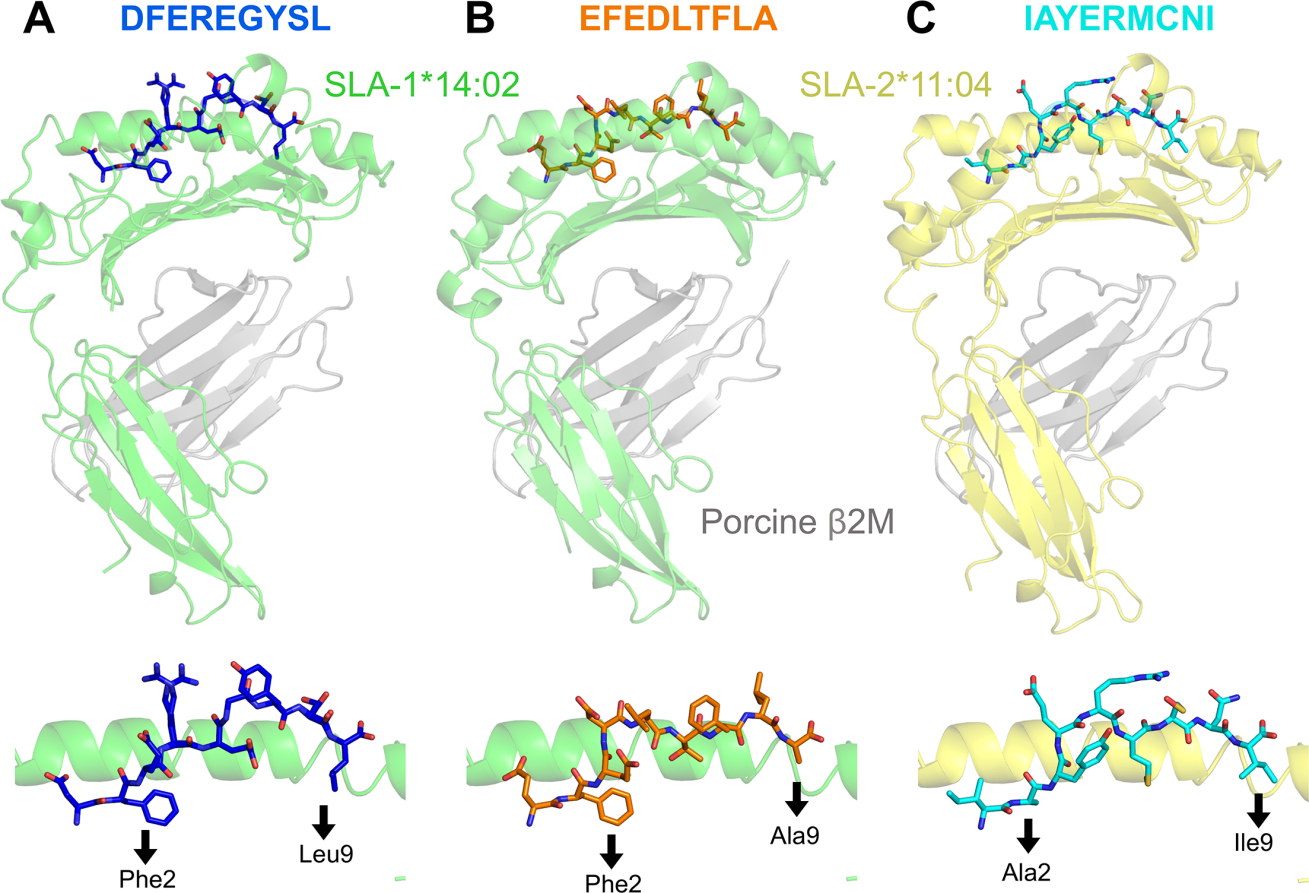
Structural overview of influenza peptides bound to SLA-1*14:02 and SLA-2*11:04. (**A**) Upper panel: SLA-1*14:02 (α1, α2 and α3 domains in green) with nucleoprotein peptide DFEREGYSL (blue) and porcine β2M (grey). Lower panel: position of DFEREGYSL within the SLA-1*14:02 binding groove and amino (position 2) and carboxyl (position 9) terminus anchor residues of the peptide are shown below the black arrows. (**B**) Same figure layout as for (A). SLA-1*14:02 (α1, α2 and α3 domains in green) with nucleoprotein peptide EFEDLTFLA (orange) and porcine β2M (grey). (**C**) Same figure layout as for (A). SLA-2*11:04 (α1, α2 and α3 domains in yellow) with nucleoprotein peptide IAYERMCNI (cyan) and porcine β2M (grey).

In order to predict whether responses to other influenza proteins are made, it was necessary to know the preferred peptide binding motifs for SLA-1*14:02 and SLA-2*11:04. Confirmation of the positions in the peptide that act as primary anchors for a given SLA is essential for generating peptide binding motifs. We therefore generated crystal structures of SLA-1*14:02-DFEREGYSL, SLA-1*14:02-EFEDLTFLA, SLA-2*11:04-IAYERMCNI and SLA-2*11:04-NGKWMRELI. Crystals and effective diffraction data were obtained for SLA-1*14:02-DFEREGYSL, SLA-1*14:02-EFEDLTFLA and SLA-2*11:04-IAYERMCNI, but not for SLA-2*11:04-NGKWMRELI. Data reduction and refinement statistics for the three pSLA-I structures are shown in **S5 Table**. The amino acids at positions (P) 2 and P9 for both DFEREGYSL and EFEDLTFLA were found to sit deep within the SLA-1*14:02 binding groove and were determined to be the primary anchor residue positions for this SLA (**Fig 7**). Residues Arg4 in DFEREGYSL and Asp4 and Leu5 in EFEDLTFLA sat prominently above the binding groove open for T-cell receptor engagement. SLA-2*11:04 also utilized P2 and P9 as the primary anchors, with Ala at P2 accommodated by a far shallower B pocket than in SLA-1*14:02 in the IAYERMCNI peptide (**Fig 7**). Residues Arg5 and Asn8 in the SLA-2*11:04-IAYERMCNI structure sit prominently above the groove open for T-cell receptor engagement. We also determined the structure of SLA-1*14:02 DFEREGYSL and EFEDLTFLA using human β2M. Human β2M is often used when making murine pMHC-I tetramers as substitution of murine β2M for the human molecule improves binding to murine CD8 [56] and can result in better performing pMHC-I tetramers. Substitution of porcine β2M for human β2M made no substantial difference to the overall pSLA-I structures (**S6 Fig**) and as no benefits were observed by use of human β2M in pSLA-I, porcine β2M was utilized for this purpose throughout.

### Determination of SLA-1*14:02 and SLA-2*11:04 binding motifs

**Fig 8:**
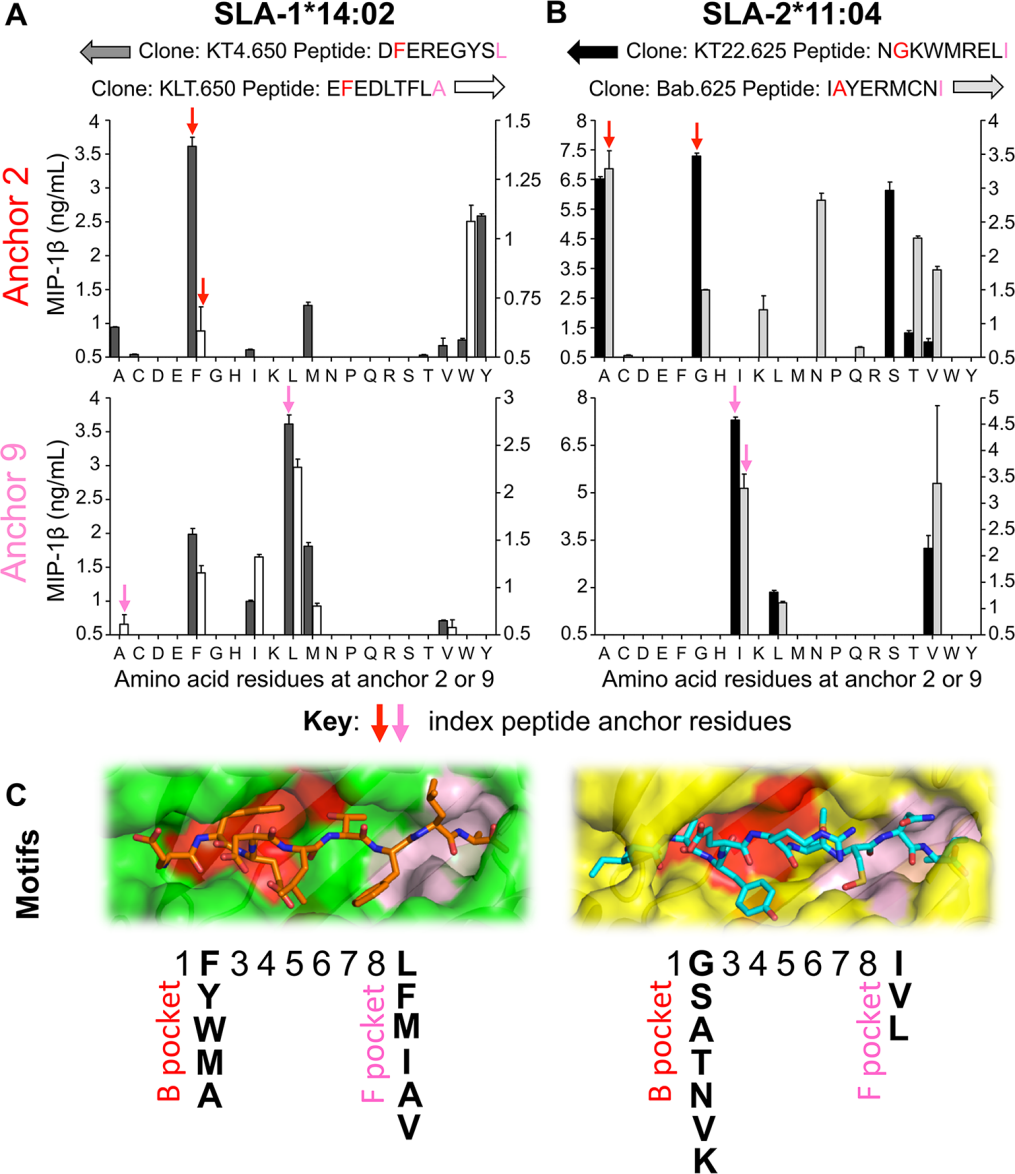
Peptide-SLA anchor residue preferences and proposed binding motifs for SLA-1*14:02 and SLA-2*11:04. **(A)** SLA-1*14:02 restricted, nucleoprotein peptide specific CD8 clones grown from Babraham pig 650 were used to define the peptide binding motif for SLA-1*14:02. Clone KT4.650 (left axis) recognizes index peptide DFEREGYSL and clone KLT.650 (right axis) index peptide EFEDLTFLA. Each of the proteogenic amino acids residues was tested at positions 2 (upper graph) and 9 (lower graph) by substitution of the index peptides. For example: D**F**EREGYSL index peptide and anchor 2 variants: D**A**/**C**/**D**/**E**/**G**/**H**/**I**/**K**/**L**/**M**/**N**/**P**/**Q**/**R**/**S**/**T**/**V**/**W**/**Y**/EREGYSL (each residue in bold tested in turn). The corresponding clone was used in peptide titration assays and ELISAs were performed to determine MIP-1β release, with data displayed for 10^−7^ M peptide. The limit of maximal detection of MIP-1β release was ~10 ng/mL, data below 0.5 ng/mL has been omitted for clarity, and mean + SEM shown. (**B**) As for (A), but using the SLA-1*11:04 restricted, nucleoprotein peptide specific clones KT22.625 (left axis, index peptide NGKWMRELI) and Bab.625 (right axis, index peptide IAYERMCNI) grown from Babraham pig 625 to define the peptide binding motif for SLA-2*11:04. Data displayed for 10^−8^ M peptide. (**C**) Binding pocket composition and proposed binding motif for SLA-1*14:02 and SLA-2*11:04 determined from the data in panels A and B. SLA-1*14:02 (green) with EFEDLTFLA (orange sticks) and SLA-2*11:04 (yellow) with IAYERMCNI (cyan sticks). Double conformers have been removed for visual clarity. B pocket is shown in red and the F pocket in pink.

Having established the primary anchor residues for both Babraham SLA-I molecules we next examined which of the 20 proteogenic amino acids could be tolerated in these positions by amino acid substitution in all four SwIV epitopes described above. Relevant T-cell clones were incubated with these peptide mutations and the wildtype ‘index’ peptides overnight and MIP-1β release was quantified (**Fig 8**). SLA-1*14:02-restricted clone KT4.650 responded best to the index amino acid, Phe, at P2 in DFEREGYSL, however KT4.650 also tolerated Ala, Met and Tyr at P2 with the latter being the second preference (**Fig 8A**). This T-cell clone also preferred the index amino acid (Leu) at P9, but also tolerated Phe and Met well and Ile and Val to a lesser extent (**Fig 8B**). The T-cell clone KLT.650 gave a stronger response to Trp than to its index anchor at P2, Phe (**Fig 8A**). Phe, Ile, Leu and Met were tolerated at P9 by this clone and preferred to the index amino acid, Ala (**Fig 8B**). These data for SLA-1*14:02 were collated to give a proposed binding motif as displayed in **Fig 8C**; [xF/Y/W/M/AxxxxxxL/F/M/I/A/V]. For SLA-2*11:04, clones KT22.625 and Bab.625 displayed a preference for their index amino acid at P2 but other residues were also tolerated (**Fig 8A**). Clone KT22.625 could also recognize residues Ala and Ser strongly at P2 and Thr and Val to a much lesser degree. In contrast, Bab.625 preferred residues Thr and Val but was also able to tolerate Asn at P2 unlike clone KT22.625. The residue tolerance at the P9 anchor was more limited for the SLA-2*11:04-restricted clones. Both clones responded well to peptides with their P9 index residue, Ile, but additionally responded to peptides with Val and Leu in this position (**Fig 8B**). The SLA-2*11:04 data were collated to give a proposed binding motif of [xG/S/A/T/N/V/KxxxxxxI/V/L] as displayed in **Fig 8C**. Application of pocket assignment as used in human HLA molecules [57] showed that for both SLA molecules, P2 and P9 of the peptide sit within B and F pockets respectively (**Fig 8C**). Pockets B and F in SLA-1*14:02 are large and deep whereas the pockets are shallower in SLA-2*11:04.

### SLA-1*14:02 and SLA-2*11:04 binding motifs allow epitope prediction from conserved influenza proteins

**Fig 9:**
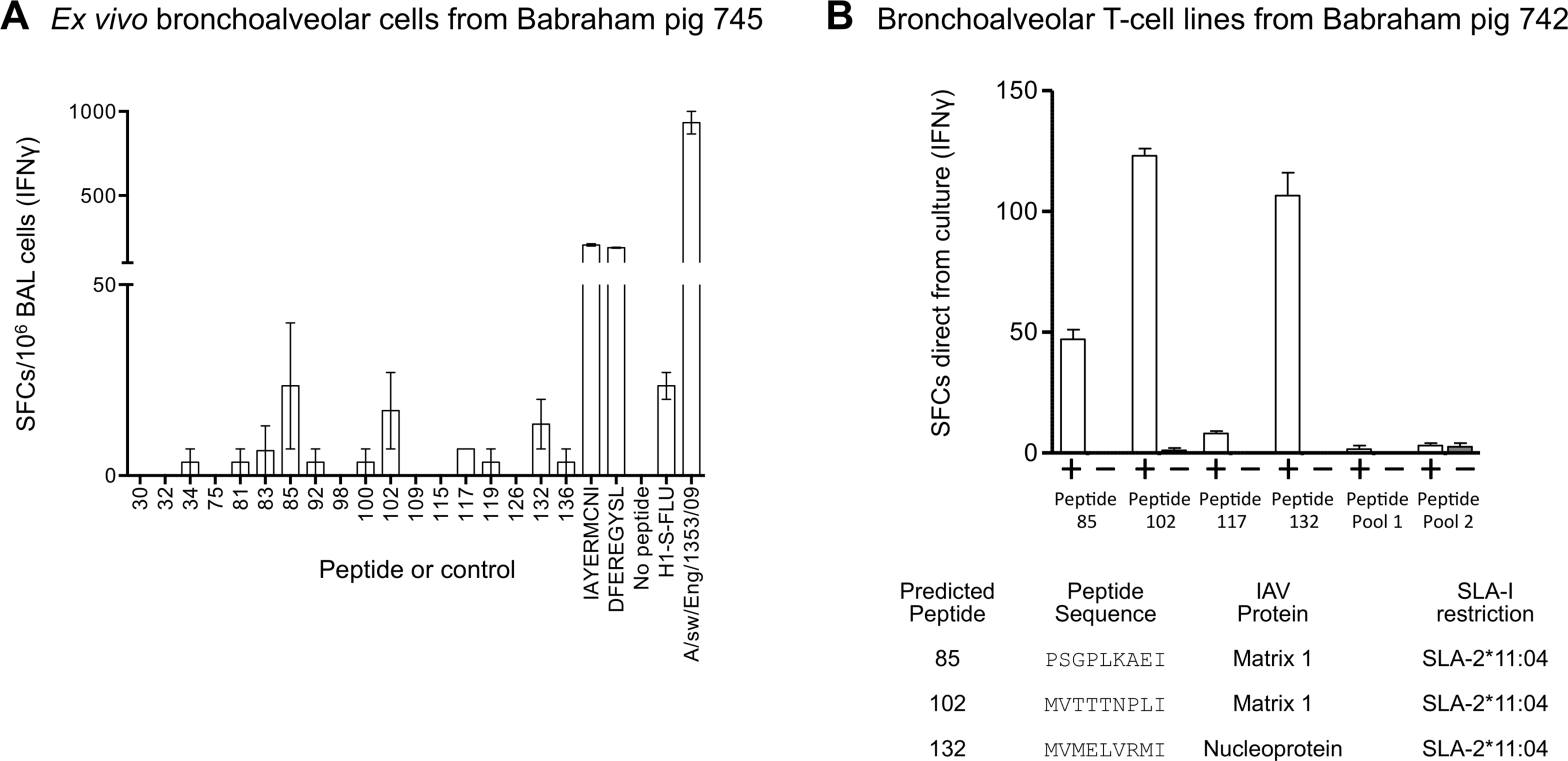
Verification of predicted influenza T-cell epitopes in bronchoalveolar from Babraham pigs infected with pandemic H1N1 swine influenza. Babraham pigs 742 and 745 were experimentally infected intranasally with H1N1 [A/sw/Eng/1353/09] and culled on day 14 post infection. Based on the binding motifs of SLA-1*14:02 and SLA-2*11:04 (**Fig 8**), predicted epitopes from matrix proteins 1 and 2, nucleoprotein, and polymerase basic proteins 1 and 2 (**S6 Table**) were tested as pooled peptides using BAL cells from pig 745 and IFNγ ELISpots (**S7 Fig**). Individual peptides highlighted from this process were then tested: (**A**) BAL cells from pig 745 were thawed and 200,000 used per well for IFNγ ELISPOT. 18 individual peptides (numbered on the x-axis) were used alongside our validated nucleoprotein epitopes, IAYERMCNI and DFEREGYSL. A no peptide and viral controls were included, and conditions performed in duplicate, scaled (X5) to 10^6^ spot forming cells (SFCs) and with the mean displayed +SEM. Babraham kidney cells were used in every well (15,000 per well) to act as antigen presenting cells. (**B**) BAL cells from pig 742 were cultured in the presence of the peptides from (A) to create six T-cell lines (labelled on the x-axis). Peptides 85, 102, 117 and 132 were used individually as they gave relatively more SFCs for the ELISPOT in A. The remaining peptides were assembled in to two pools; pool 1 consisted of peptides 30, 32, 34, 75, 81, 83, 92; and pool 2 contained peptides 98, 100, 109, 115, 126, 136 (sequences in **S6 Table**). After two weeks, the lines were taken straight from culture for IFNγ ELISPOT and incubated with 10^−5^ M of the peptide(s) used to generate the line (+) or with no peptide (−). The actual number of SFCs is displayed as mean +SEM. The table shows the peptide sequence, the protein of origin and SLA-I restriction of the three epitopes that gave robust responses.

To preliminary test whether the novel binding motifs for SLA-1*14:02 and SLA-2*11:04 could predict other epitopes we scanned five conserved influenza proteins; matrix proteins 1 and 2; polymerase basic proteins 1 and 2; and NP; with the Babraham SLA-I motifs generated above using ‘Motif Scan’ available at www.hiv.lanl.gov. The scan generated 292 predicted potential epitopes (**S6 Table**). These peptides were synthesized, assembled into a peptide matrix and were incubated with BAL cells from a swine influenza infected pig (745) *ex vivo* and IFNγ release was measured by ELISpot (**S7 Fig**). This initial scan identified 18 peptides for individual testing which were incubated with pig 745 BAL cells alongside our validated influenza epitopes, IAYERMCNI and DFEREGYSL, as positive controls (**Fig 9A**). Strong responses were observed to epitopes IAYERMCNI and DFEREGYSL with 206 and 190 SFC per 10^6^ BAL cells, respectively. Responses to the 18 predicted peptides were much lower (<5 SFC) or absent *ex vivo* (**Fig 9A**). To increase any responses present, BAL cells were cultured with the most promising peptides from the initial analysis. Accordingly, BAL cells were primed with four peptides individually and the remaining peptides were combined into two pools due to limited availability of BAL samples. After two weeks of culture, the primed lines were incubated with and without their respective peptide(s) and IFNγ release measured (**Fig 9B**). This approach identified clear responses to three peptides; (85) PSGPLKAEI; (102) MVTTTNPLI; (132) MVMELVRMI, the former two are derived from viral protein M1 and the latter from NP. All three of these peptides are predicted to be restricted by SLA-2*11:04. These data demonstrate that T-cell responses can be generated to other SwIV-derived peptides predicted from the SLA binding motifs we have generated here.

## Discussion

The anatomical and physiological parallels between humans and pigs make the pig a valuable non-primate model for clinical research. The Babraham large white inbred pig line is >85% genetically identical and matched for all SLA, making it particularly suited for immunological studies [24]. Unfortunately, immunological tools such as robust SLA peptide binding motifs, defined T-cell epitopes and multimer technology for pigs have lagged well behind that available in humans or experimental mice. This study aimed to close that gap and advance the Babraham pigs as an immunological model to study swine and human diseases. Specifically, we examined the CD8 T-cell responses induced by the BPIV candidate, S-FLU [25–27], a non-pathogenic pseudotyped Influenza virus, which has its HA-signal sequence suppressed, preventing it from replicating within the host. S-FLU has shown protective efficacy, in the complete absence of, or presence of low levels of neutralizing antibodies, following homologous and heterologous influenza challenge in both mice and ferrets and prevented airborne transmission in the latter [25,26]. S-FLU has also been shown to induce local lung T-cell responses and reduce viral load in outbred pigs [27].

Using a non-assumptive approach of overlapping peptides spanning the whole protein sequence of PR8 strain NP, we generated influenza-specific CD8β+ T-cell lines and defined four new SwIV epitopes in the Babraham pigs. Refolding of these epitopes with the protein sequences of the extracellular domains of the Babraham SLA-I alleles, SLA-1*14:02 and SLA-2*11:04, showed that two of the peptides, DFEREGYSL and EFEDLTFLA, bound to SLA-1*14:02 while the other two, IAYERMCNI and NGKWMRELI, bound to SLA-2*11:04.

Others have identified putative SwIV epitopes, restricted by one of the most commonly occurring SLA in outbred pigs, SLA-1*0401 [22] or SLA-1*0702 [23], using an *in silico* predictive algorithm. Another study, using the immunoinformatics tool Pig Matrix, identified a number of SLA-1 epitopes highly conserved in seven representative SwIV strains in the US [51]. While these studies were performed in outbred pigs, a recent study in genetically defined NIH mini pigs used tetramers to characterize the specificity of cytotoxic T-cells following multiple inoculations with adenovectored foot-and-mouth disease vaccines [58]. These previous studies have not had the luxury of having T-cell lines and clones with which to optimize T-cell staining protocols. Here, we grew the first pig T-cell clones that we are aware of and used these monoclonal T-cell populations to optimize pSLA-I staining protocols. The Babraham pig forms an ideal model for immunological studies as it carries just one MHC haplotype. The resulting match of SLA class I and II alleles in these animals allows adoptive transfer of immune cells between individuals and means that fewer numbers of animals per group can be used for immunological studies compared with outbred pigs. The Babraham MHC-I molecules, SLA-1*14:02 and SLA-2*11:04, are not known to occur widely in outbred domestic pigs and it remains to be seen whether other, more common, SLA-I molecules present widely overlapping peptide repertoires. The vast variability of HLA-I alleles in the human population (>10,000 different alleles described to date) distils down to just a dozen “supertypes” [59] based on the molecular structures of binding pockets. It is not yet possible to define porcine supertypes or establish how widely the new epitopes we describe will be presented in the outbred livestock population.

The availability of pig T-cell clones allowed us to define minimal epitopes. Our laboratory has a long track record of optimizing pHLA multimer staining [35,37,38,60]. We have recently determined that standard staining protocols can fail to detect fully functional anti-viral T-cells that can be detected with an optimized procedure that utilizes PKI and antibody cross-linking [61]. We showed the use of PKI and anti-fluorochrome Ab, applied to improve pHLA multimer staining in humans (reviewed in [36]), also increased the percentage of antigen specific T-cells detected in pigs. We therefore recommend application of these techniques to pig tetramer studies to obtain clearer staining and optimal T-cell detection. Enhancing T-cell detection with pSLA multimers was particularly relevant here as the majority of our samples were taken two weeks after vaccination or infection when T-cells were likely to be relatively activated with low expression of T-cell receptor making them more difficult to detect using conventional staining techniques. In all previous studies in pigs, the tetramers were tested using PBMC samples. For the first time, we have analyzed responses in local tissues. T-cells recognizing the four NP epitopes were detected in the PBMC, BAL and TBLN of the simultaneously mucosally and parenterally immunized pigs. Although one vaccine was delivered intramuscularly with adjuvant and the H5N1 S-FLU intranasally, the responding T-cells were concentrated in the BAL and TBLN compared to the PBMC. CD8+ T-cell responses in BAL were between 3- and >100-fold higher than those observed in equivalent PBMC.

More importantly, 3-out-of-the-4 epitopes were recognized by T-cells in pigs immunized with H1N1 S-FLU by aerosol on its own. Additionally, nearly 40% of the total CD8+ T-cells in the BAL in one of the pigs responded to these three epitopes while almost a quarter of CD8+ T-cells were specific for a single epitope. These findings are consistent with IAV infection in mice where over 60% of the CD8+ T-cells in the very high level of immune cell infiltrate in BAL can be specific for a single IAV epitope [62,63]. Immunization of outbred pigs with H1N1 S-FLU by aerosol significantly reduced nasal and lung viral titres after homologous challenge in previous experiments, in the absence of neutralizing antibodies [27]. These results indicate that highly focused T-cell responses to conserved epitopes show protective efficacy after aerosol delivery of this BPIV candidate. Similar results showing a superior protection of aerosol delivery compared to other mucosal routes have been shown with adenoviral vectored tuberculosis or Ebola vaccines in non-human primates [64,65]. Previous studies in outbred pigs infected intratracheally with H1N2 SwIV detected virus specific IFNγ-producing CD8 T-cells in the lungs at frequencies up to 30 times higher than in PBMC and TBLN [66]. Here we show that two out of the four immunodominant epitopes we have identified in simultaneously immunized animals are recognized by T-cells in the BAL of SwIV infected animals. To our knowledge this is the first analysis of local T-cell responses in BAL by pSLA multimers after aerosol immunization or nasal infection with SwIV and demonstrates the magnitude, specificity and focussing of these cellular immune responses.

To date, just two pSLA-I structures have been deposited in the public database [45,52], compared to several hundred pHLA structures. Here we successfully generated high-resolution structures for three of the four NP epitopes we initially discovered, SLA-1*14:02-DFEREGYSL, SLA-1*14:02-EFEDLTFLA and SLA-2*11:04-IAYERMCNI. In each case, these structures allowed identification of the primary anchors for SLA-1*14:02 and SLA-2*11:04 as P2 and the C-terminus. This is consistent with previously published pSLA-I structures where P2 and the C-terminus can be seen sitting deep within the groove and acting as primary anchors for SLA-1*0401 [45] and SLA-3*hs0202 [52]. Amino acid substitution at these positions in all four epitopes was used to establish which amino acids could be tolerated in these positions and build a peptide binding motif for each allele. The motif for SLA-1*14:02, [xF/Y/W/M/AxxxxxxL/F/M/I/A/V], conforms to that determined in preliminary studies using elution of self-peptides which produced a more stringent motif of [x-Y/F/A-xxxxxx-L/I] (http://randd.defra.gov.uk/Document.aspx?Document=SE1509_3351_FRP.doc). A different motif, [xG/S/A/T/N/V/KxxxxxxI/V/L] was identified for SLA-2*11:04. The preferences for these residues became understandable when the pockets of the SLA binding groove were structurally examined. P2 is accommodated by the B pocket which is large and deep in SLA-1*1402 allowing it to tolerate large aromatic residues whereas in SLA-2*11:04 the pocket is shallower and less selective. The F pocket accommodates the C-terminus (P9), again in SLA-1*1402 it is larger and can tolerate larger residues (F and M) whereas in SLA-2*11:04 it is shallower with limited tolerance.

We next made use of the SLA binding motifs to see if we could identify further SwIV epitopes by *in silico* prediction as a proof of concept. Predicted peptides from the matrix proteins 1 and 2, NP, and polymerase binding protein 1 and 2 were tested on BAL samples from H1N1 infected pigs both *ex vivo* and following *in vitro* priming. These preliminary studies identified three potential new subdominant influenza epitopes restricted to SLA-2*11:04; PSGPLKAEI (Matrix 1), MVTTTNPLI (Matrix 1) and MVMELVRMI (NP). These all contain a C-terminal isoleucine residue, as with the previous epitopes, thereby supporting the strong preference for this residue at this position. Additionally, the three epitopes contain different P2 residues than IAYERMCNI and NGKWMRELI reinforcing the validity of our epitope prediction motif. Further work may be required in order to validate these putative epitopes as the responses are much smaller than those identified for the immunodominant IAYERMCNI and NGKWMRELI epitopes. Removal of these immunodominant responses by mutation might then allow subdominant responses to be identified more cleanly but was beyond the scope of our project. Future studies could extend this approach to other influenza vaccination or infection settings and to other porcine disease studies. It will also be important to use a refined motif that allows for selection of 10-mer peptides as extrapolation from human systems suggests that up to 20% of SLA-I epitopes might be of longer length [49]. Overall, the successful prediction of the three further influenza-derived peptide sequences that could stimulate T-cell responses indicates that the motifs generated in this study will be useful for identifying epitopes in other diseases.

In summary, this study constitutes a substantial advance in the immune toolkit available for studying influenza responses in swine. In addition to establishing robust pig T-cell culture for the first time we have identified SLA binding motifs, influenza-derived SLA-I epitopes and established pSLA-I tetramer staining in the Babraham inbred pig model. We provide epitope prediction motifs for both SLA-I molecules from these pigs. These tools enabled us to characterize the local lung T-cell immune response to a BPIV candidate, S-FLU and following SwIV infection in pigs. For the first time we show the specificity, magnitude and longevity of T-cell response after aerosol delivery of vaccine and the high immunogenicity and efficacy of this method of immunization. This toolkit will be used in future to assess vaccine efficacy and immune correlates of protection. Furthermore, these advances can be applied to other economically important diseases in swine, such as foot-and-mouth disease, Nipah virus and African swine fever. Collectively, this work augments the establishment of pigs as an important model of human influenza infection and further the use of pigs as models for other clinically-relevant human diseases. Indeed, we are currently exploring the potential use of non-biologic T-cell ligands for IAV vaccination [67] in the Babraham model.

## Supporting information

S1 Table Sex, weight, SLA-I typing and age of Babraham pigs used in experiments. (TIF)

S2 Table Sequence conservation of nucleoprotein epitopes amongst influenza viral strains utilised in this study. (TIFF)

S3 Table Percentage of CD3^+^, CD8β+ and CD8β/CD4 double + cells in Babraham pigs used in this study. (TIFF)

S4 Table List of overlapping peptides of nucleoprotein from PR8. (TIFF)

S5 Table Data reduction and refinement statistics. (TIFF)

S6 Table SLA-1*14:02 and SLA-2*11:04 predicted epitopes for influenza viral proteins Matrix (M) 1, Matrix 2, nucleoprotein (NP), polymerase binding protein (PB) 1 and polymerase binding protein 2. (TIFF)

S1 Fig Cell surface detection of TNF following activation of Babraham pig T-cells and gating strategy for pSLA tetramer staining of blood, bronchoalveolar and tracheobronchial lymph node samples. (**A**) PBMCs incubated +/- phytohaemagglutinin in the presence of TNF processing inhibitor-0 (TAPI-0) allowing detection of cell surface bound TNF with anti-TNF antibody. Gated: viable lymphocytes and displayed as CD3 cells versus TNF. Percentage of gated cells displayed. (**B**) Purified CD8β cells stimulated with peptide for 2 weeks followed by reactivation +/- peptide in the presence of TNF processing inhibitor-0 (TAPI-0) as in A. Gating Viable lymphocytes displaying forward scatter (FSC) versus TNF. Percentage of gated cells displayed. (**C**) Representative peripheral blood mononuclear cell sample is displayed from Babraham pig 625. Cells were gated sequentially; Gate 1: for size and structure (lymphocyte gate); Gate 2: single cells; Gate 3: viable (vivid^neg^) CD3^+^ CD14^neg^ cells; Gate 4: CD4^+^ and CD8β^+^. The gating strategy removes cells that may bind tetramers non-specifically (dead, CD14^+^, CD8β^neg^/CD4^neg^). (**D**) Gated cells were then displayed as CD8β expression versus pSLA tetramer staining. The CD8β^+^ T-cells are the subset of interest (blue gate). CD4^+^ cells were used as an irrelevant T-cell subset (green gate) to assess the degree of background staining (orange gate) relative to influenza tetramer staining (red gate). Additionally (left flow plot), irrelevant peptides refolded with SLA-1 or -2 of the Babraham were used as ‘control/irrelevant’ tetramers alongside the influenza tetramers, to assess the background staining (purple gate) of the CD8β subset (blue gate). Of all the Babraham pigs used for staining, 100% of the influenza tetramer^+^ cells were CD8β^+^ with less than 1% also staining for CD4.
(TIFF)

S2 Fig Generation of influenza-specific CD8β T-cell lines from Babraham pig 625 simultaneously immunized with H5N1-S-FLU and Sp/Sw H1N1. (**A**) Purification of CD8β cells using an anti-CD8β unconjugated antibody (Ab), a secondary PE conjugated Ab and anti-PE magnetic microbeads. The dot plot displays all viable cells prior to magnetic enrichment showing CD8β staining. The histogram shows the pre-sorted (black) and post sorted cells; negative fraction (grey) and CD8β^+^ fraction (blue), with percentages shown for the gated cells. The purified CD8β cells from pig 625 were used to create T-cell lines by incubation with pooled or individual overlapping peptides from the nuceloprotein of S-FLU (PR8). Irradiated CD8β^neg^ cells from pig 650 were used to present peptide. (**B**) A T-cell line generated by incubation with peptide pool A. Intracellular staining was performed for TNF following incubation with DMSO (no peptide), peptide pool A or individual peptides from pool A, with only positive responses being displayed. The percentage of cells responding to peptide are gated and shown in red. The blue gate and percentage shows the proportion of the CD8β^neg^ cells post 14 d of incubation. (**C&D**) Using the same approach as in (A) for a T-cell line generated to pool B, and later mapped to individual peptides 36 and 37. (**E**) Using the same approach as in (A) for a T-cell line generated for peptide pool C. Gating strategy: lymphocytes and viability (Vivid^neg^).
(TIFF)

S3 Fig Generation of influenza-specific CD8β T-cell lines from Babraham pig 650 simultaneously immunized with H5N1-S-FLU and Sp/Sw H1N1. Purified CD8β cells from pig 650 were used to create T-cell lines by incubation with pooled or individual overlapping peptides from the nucleoprotein of S-FLU (PR8). Irradiated CD8β^neg^ cells from pig 650 were used to present peptide. (**A**) A T-cell line generated by incubation with peptide pool A. Intracellular staining was performed for TNF following incubation with DMSO (no peptide), peptide pool A or individual peptides from pool A, with only positive responses being displayed. The percentage of cells responding to peptide are gated and shown in red. The blue gate and percentage shows the proportion of the CD8β^neg^ cells that are present in the line 14 d post being set-up. (**B**) Using the same approach as in (A) for a T-cell line generated for peptide pool C. Gating strategy: lymphocytes and viability (Vivid^neg^).
(TIFF)

S4 Fig Testing anti-CD8β antibody clones. (**A**) Antibody (Ab) clones PG164A and PPT23 specific for pig cytotoxic T-cells (bind CD8β) were tested on a Large White/Landrace cross and Babraham pig. Cells were gated on viable CD3^+^ T-cells and displayed as CD8β versus CD4 expression. Percentages are shown for the CD8β^+^, CD8β^+^/CD4^+^ and CD4^+^ populations. Similar proportions of cells were stained for each of the antibody clones. (**B**) The CD8β^+^/CD4^+^ population ranged between 0.04–1.25% of viable CD3+ T-cells, with a mean of 0.41%. Representative plots from an experimental Babraham pig (625) showing all events, as needed for tetramer analysis, and the same number of events (20,000) as the plots in A. (**C**) Using the same approach as in A, displaying CD8β versus CD8α staining. Both anti-CD8β antibody clones clearly stained CD8β^+^/CD8α^+^ T-cells that were distinct from CD8β^neg^/CD8α^+^ T-cells. Percentages are shown for CD8β^+^/CD8α^+^ and CD8β^neg^/CD8α^+^ populations.
(TIFF)

S5 Fig Optimization of pSLA tetramer staining protocols using protein kinase inhibitor and anti-fluorochrome antibody. Nucleoprotein or irrelevant peptide SLA tetramers (PE conjugated) were used to stain peripheral blood mononuclear cells from Babraham pig 650, either without (standard protocol) or with (optimized protocol) the addition of protein kinase inhibitor Dasatinib and anti-fluorochrome-PE antibody. The sequences of the nucleoprotein peptides and their restriction are shown. A self-eluted peptide derived from ferritin (EYLFDKHTL) was used as an irrelevant tetramer. The percentage of tetramer^+^ cells of CD8β^+^ cells is displayed in red. Gating strategy: lymphocytes, single cells, viability (Vivid^neg^)/CD3^+^/CD14^neg^ then CD8β^+^/CD4^+^ and displayed as CD8β versus tetramer (**S1 Fig**).
(TIFF)

S6 Fig Comparison of SLA-1*14:02 with nucleoprotein peptides DFEREGYSL and EFEDLTFLA using either porcine or human β2M. (**A**) The overall structure of SLA-1*14:02 binding nucleoprotein peptide DFEREGYSL refolded with either porcine β2M (green and grey) or human β2M (purple). (**B**) Porcine and human β2M from A compared only. (**C**) The overall structure of SLA-1*14:02 binding nucleoprotein peptide EFEDLTFLA refolded with either porcine β2M (green and grey) or human β2M (orange). (**D**) Porcine and human β2M from C compared only. Root-means-square (RMS) deviations are displayed for each comparison.
(TIFF)

S7 Fig Strategy and preliminary data for defining new influenza epitopes for Babrham pigs. (**A**) A peptide pool matrix comprising predicted SLA-1*14:02 and SLA-2*11:04 peptides from nucleoprotein (NP), Matrix (M) 1 and 2, and polymerase basic proteins (PB) 1 and 2 (peptide sequences present in **S6 Table**). Each peptide was present in 2 of the pools allowing rapid *ex vivo* screening of all peptides with a limited number of cells. Peptides IAYERMCNI, NGKWMRELI, DFEREGYSL and EFEDLTFLA defined in previous experiments were also amongst the matrix. Dark grey indicates a strong ELISPOT response (**B**) to the respective pool whereas the light grey is for relatively lower responses (**B**). The intersect between peptide pools of the matrix that elicited a response indicated the individual peptides (boxed) (**C**) to be tested on Babraham samples (**Fig 9**). The restricting SLA for each of the selected peptides is shown.
(TIFF)
