# Supplementary figures and images for: Induction of influenza-specific local CD8 T-cells in the respiratory tract after aerosol delivery of vaccine antigen or virus in the Babraham inbred pig
Source: PLoS Pathog. 2018 May 17;14(5):e1007017. doi: 10.1371/journal.ppat.1007017 (PMC5957346; doi:10.1371/journal.ppat.1007017)

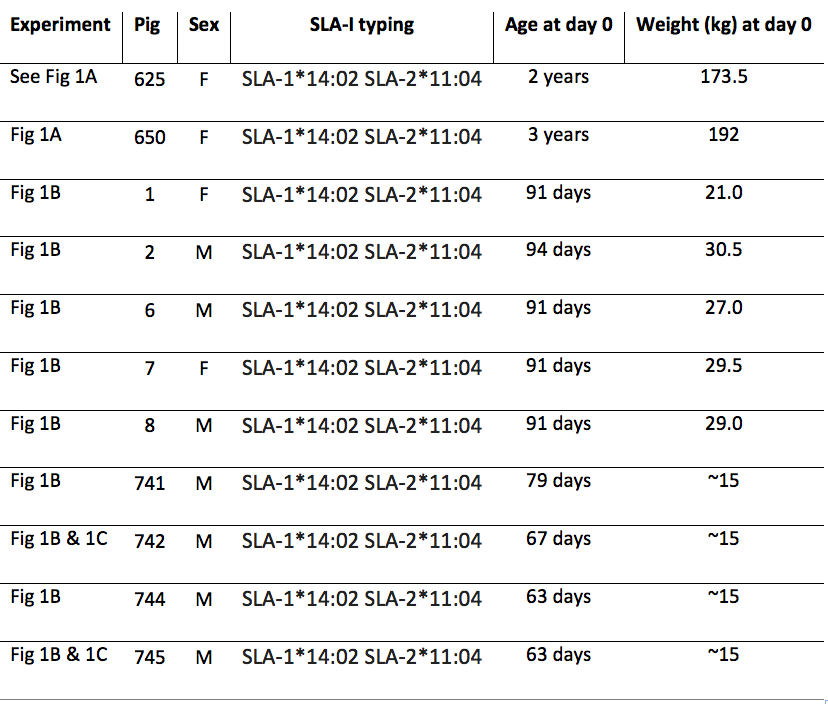

Supplement: S1 Table — (TIF) [file ppat.1007017.s001.tif]

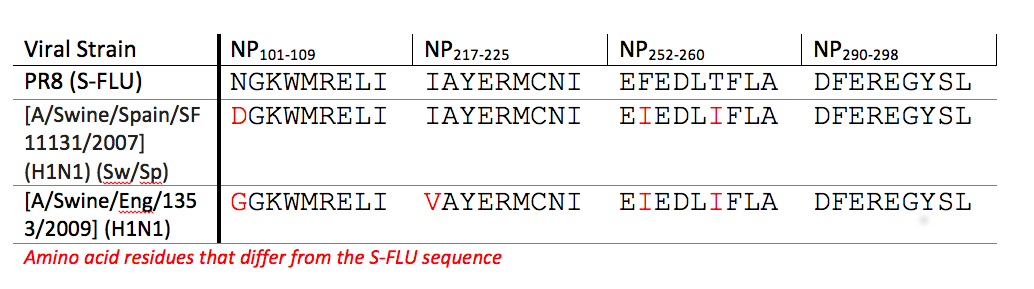

Supplement: S2 Table — (TIFF) [file ppat.1007017.s002.tiff]

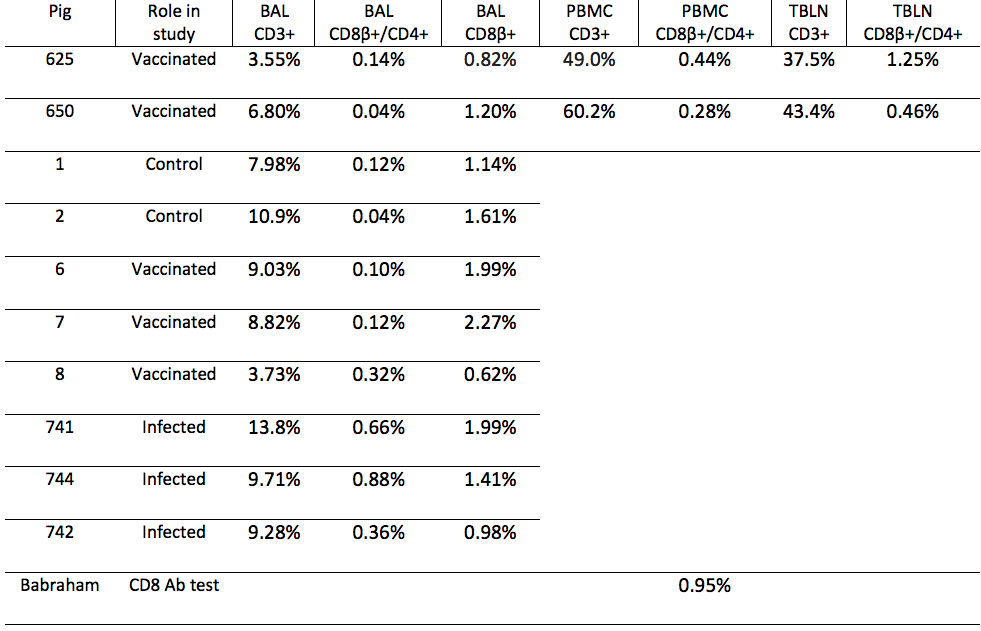

Supplement: S3 Table — (TIFF) [file ppat.1007017.s003.tiff]

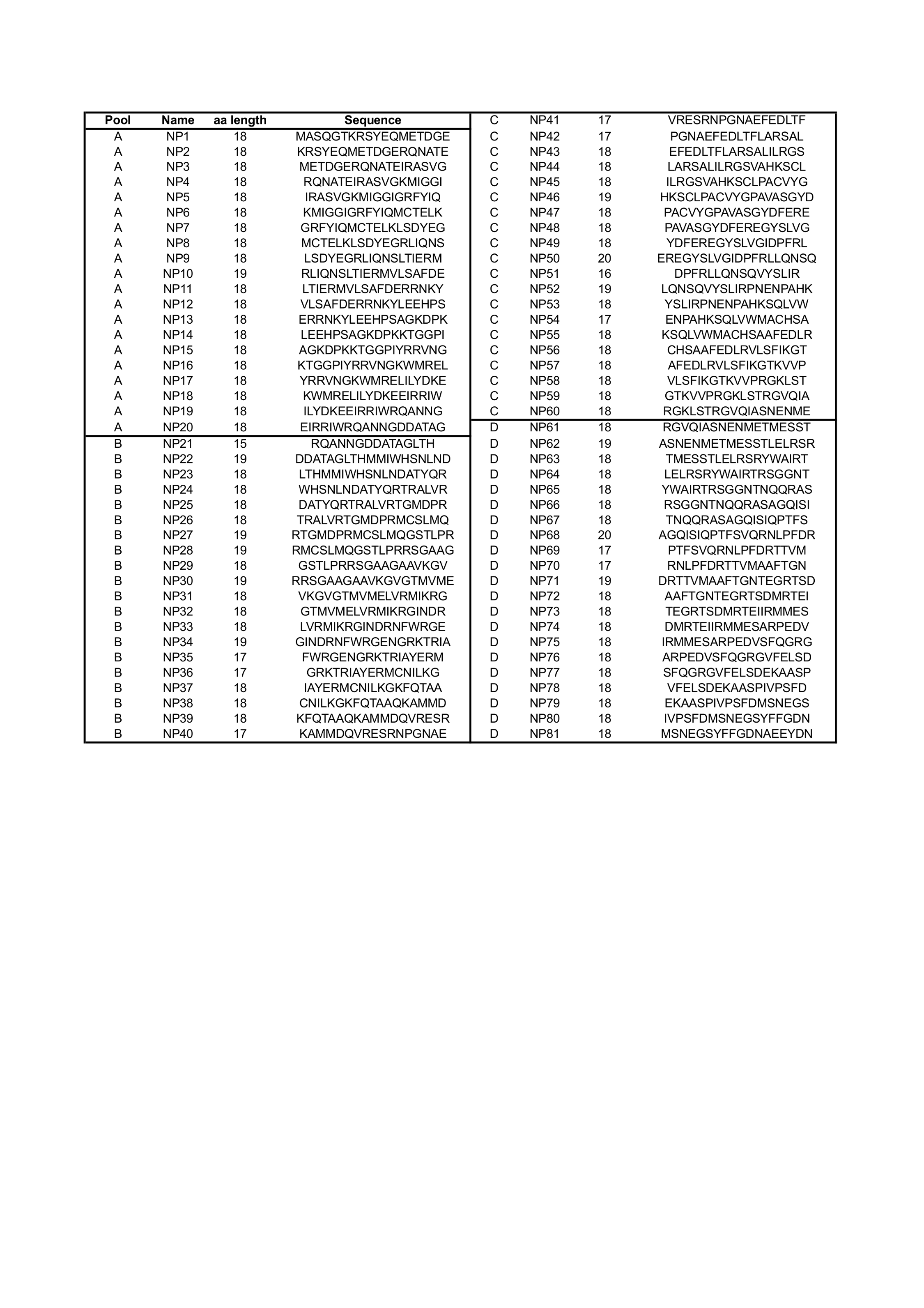

Supplement: S4 Table — (TIFF) [file ppat.1007017.s004.tiff]

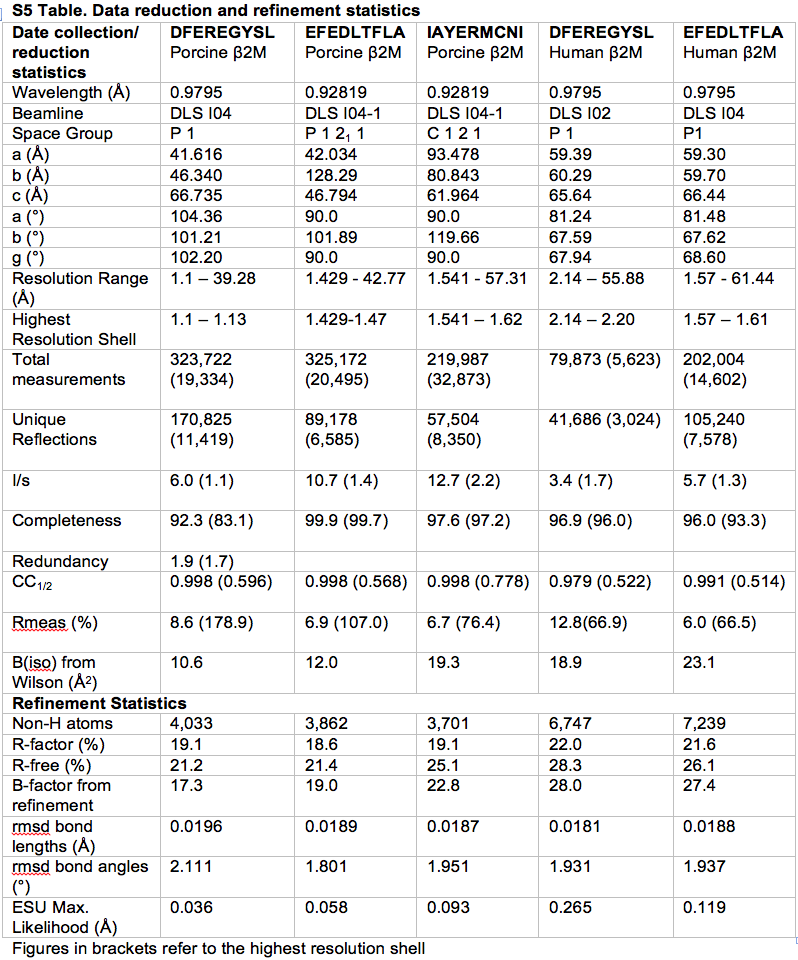

Supplement: S5 Table — (TIFF) [file ppat.1007017.s005.tiff]

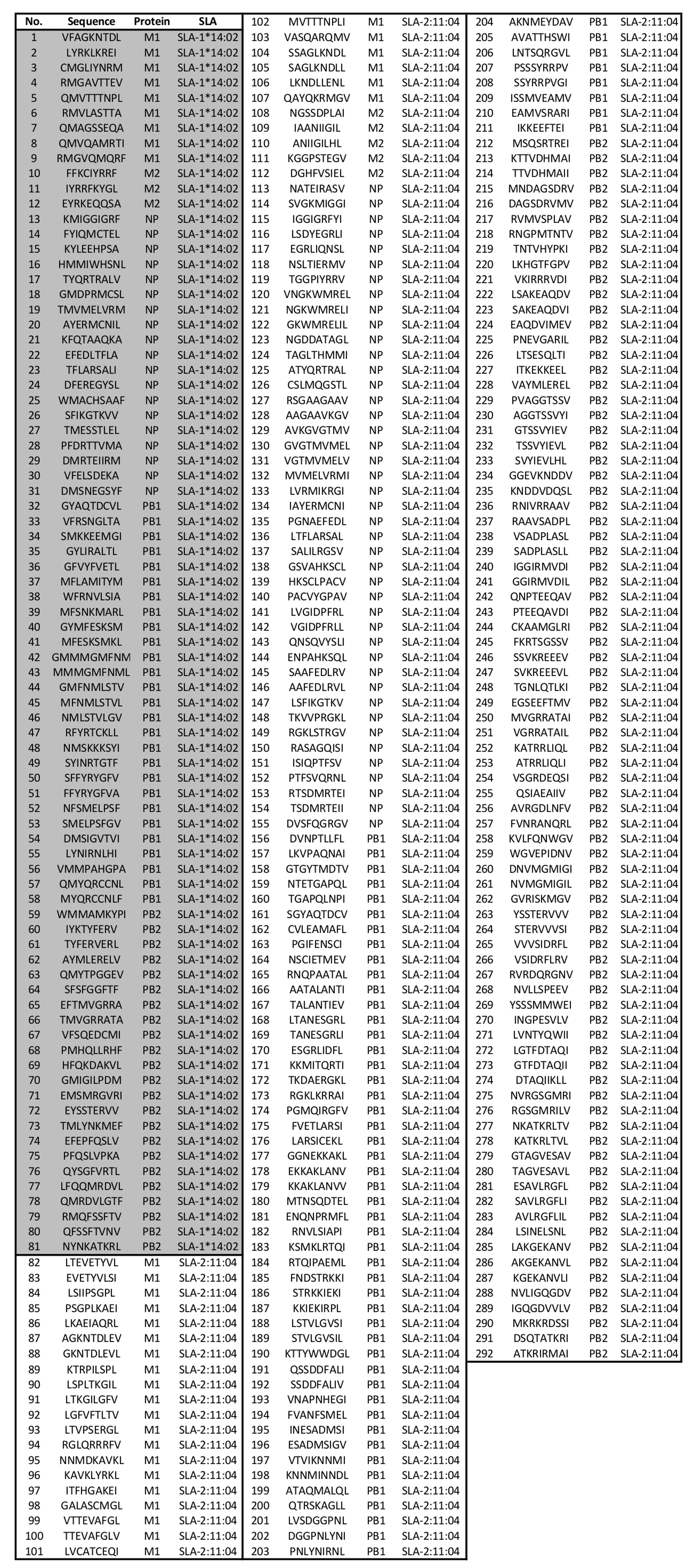

Supplement: S6 Table — (TIFF) [file ppat.1007017.s006.tiff]

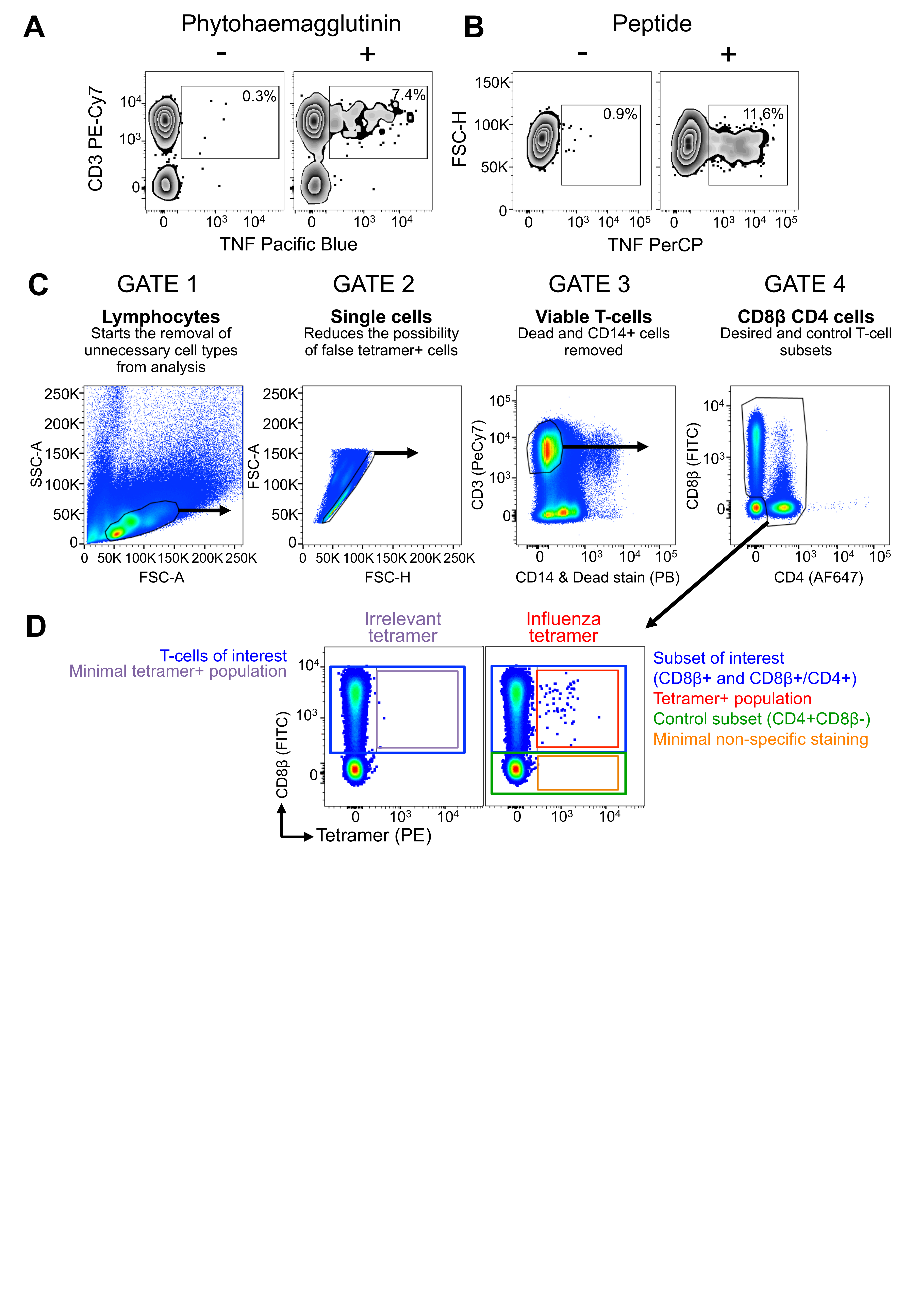

Supplement: S1 Fig — (A) PBMCs incubated +/- phytohaemagglutinin in the presence of TNF processing inhibitor-0 (TAPI-0) allowing detection of cell surface bound TNF with anti-TNF antibody. Gated: viable lymphocytes and displayed as CD3 cells versus TNF. Percentage of gated cells displayed. (B) Purified CD8β cells stimulated with peptide for 2 weeks followed by reactivation +/- peptide in the presence of TNF processing inhibitor-0 (TAPI-0) as in A. Gating Viable lymphocytes displaying forward scatter (FSC) versus TNF. Percentage of gated cells displayed. (C) Representative peripheral blood mononuclear cell sample is displayed from Babraham pig 625. Cells were gated sequentially; Gate 1: for size and structure (lymphocyte gate); Gate 2: single cells; Gate 3: viable (vividneg) CD3+ CD14neg cells; Gate 4: CD4+ and CD8β+. The gating strategy removes cells that may bind tetramers non-specifically (dead, CD14+, CD8βneg/CD4neg). (D) Gated cells were then displayed as CD8β expression versus pSLA tetramer staining. The CD8β+ T-cells are the subset of interest (blue gate). CD4+ cells were used as an irrelevant T-cell subset (green gate) to assess the degree of background staining (orange gate) relative to influenza tetramer staining (red gate). Additionally (left flow plot), irrelevant peptides refolded with SLA-1 or -2 of the Babraham were used as ‘control/irrelevant’ tetramers alongside the influenza tetramers, to assess the background staining (purple gate) of the CD8β subset (blue gate). Of all the Babraham pigs used for staining, 100% of the influenza tetramer+ cells were CD8β+ with less than 1% also staining for CD4. (TIFF) [file ppat.1007017.s007.tiff]

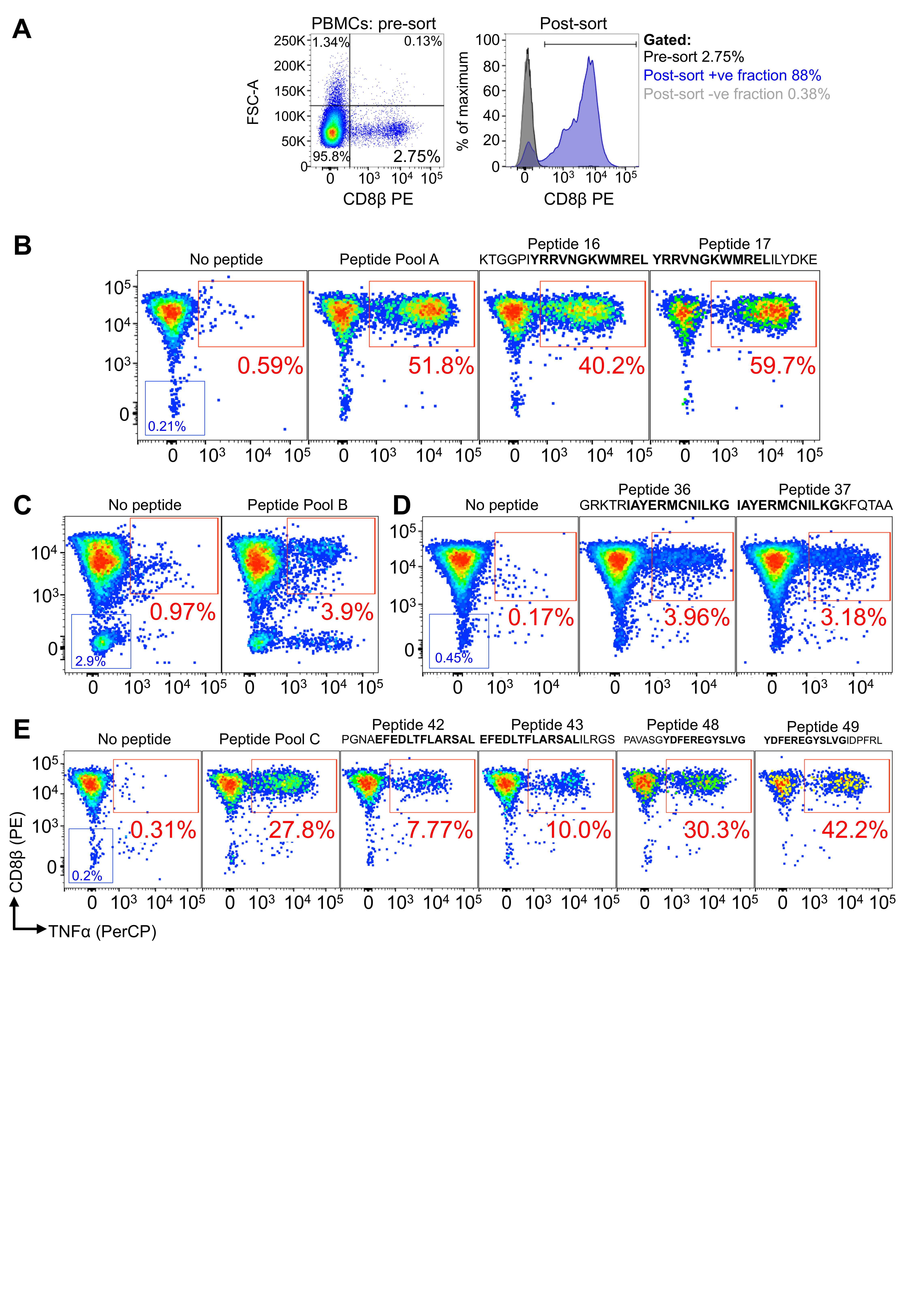

Supplement: S2 Fig — (A) Purification of CD8β cells using an anti-CD8β unconjugated antibody (Ab), a secondary PE conjugated Ab and anti-PE magnetic microbeads. The dot plot displays all viable cells prior to magnetic enrichment showing CD8β staining. The histogram shows the pre-sorted (black) and post sorted cells; negative fraction (grey) and CD8β+ fraction (blue), with percentages shown for the gated cells. The purified CD8β cells from pig 625 were used to create T-cell lines by incubation with pooled or individual overlapping peptides from the nuceloprotein of S-FLU (PR8). Irradiated CD8βneg cells from pig 650 were used to present peptide. (B) A T-cell line generated by incubation with peptide pool A. Intracellular staining was performed for TNF following incubation with DMSO (no peptide), peptide pool A or individual peptides from pool A, with only positive responses being displayed. The percentage of cells responding to peptide are gated and shown in red. The blue gate and percentage shows the proportion of the CD8βneg cells post 14 d of incubation. (C&D) Using the same approach as in (A) for a T-cell line generated to pool B, and later mapped to individual peptides 36 and 37. (E) Using the same approach as in (A) for a T-cell line generated for peptide pool C. Gating strategy: lymphocytes and viability (Vividneg). (TIFF) [file ppat.1007017.s008.tiff]

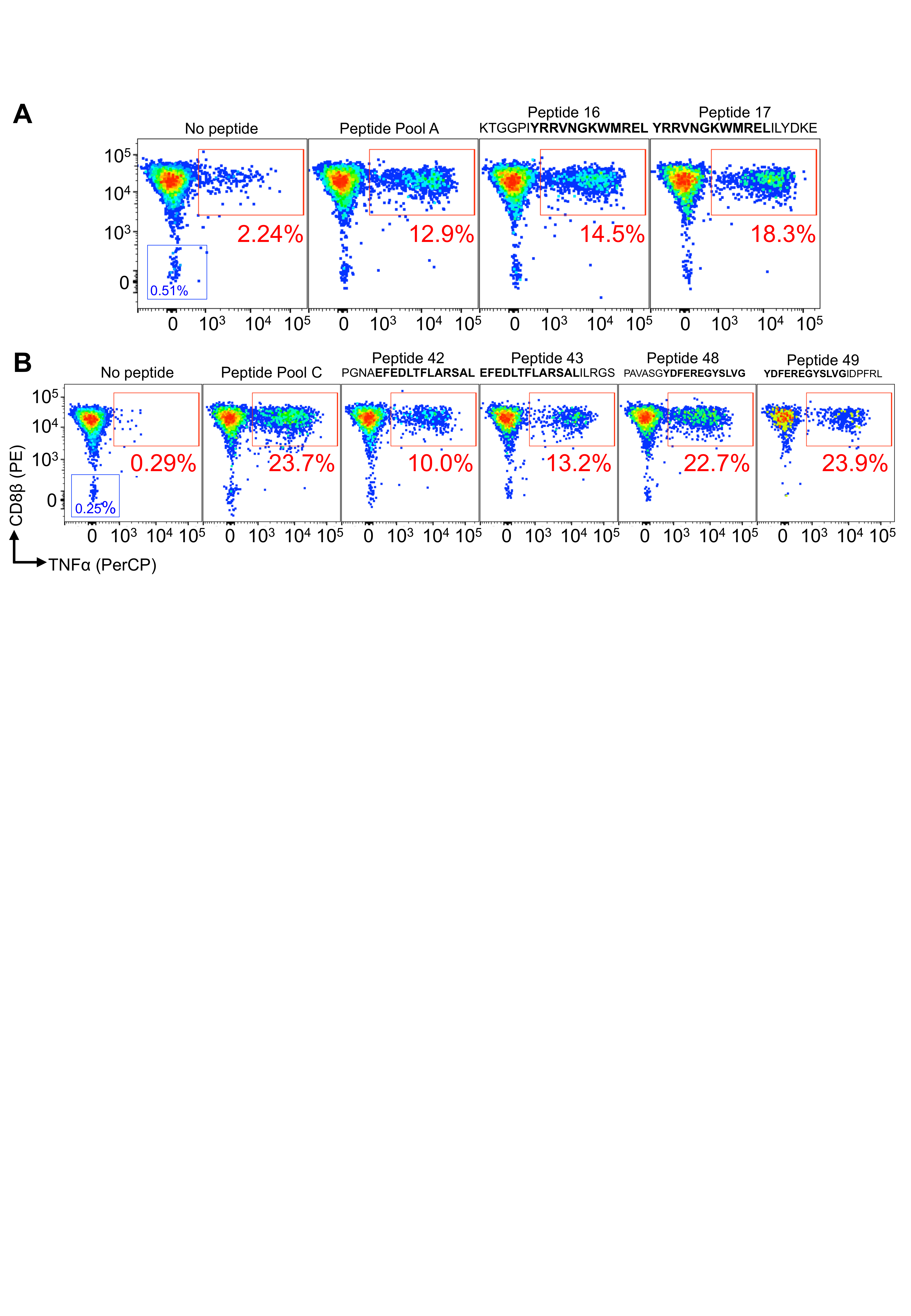

Supplement: S3 Fig — Purified CD8β cells from pig 650 were used to create T-cell lines by incubation with pooled or individual overlapping peptides from the nucleoprotein of S-FLU (PR8). Irradiated CD8βneg cells from pig 650 were used to present peptide. (A) A T-cell line generated by incubation with peptide pool A. Intracellular staining was performed for TNF following incubation with DMSO (no peptide), peptide pool A or individual peptides from pool A, with only positive responses being displayed. The percentage of cells responding to peptide are gated and shown in red. The blue gate and percentage shows the proportion of the CD8βneg cells that are present in the line 14 d post being set-up. (B) Using the same approach as in (A) for a T-cell line generated for peptide pool C. Gating strategy: lymphocytes and viability (Vividneg). (TIFF) [file ppat.1007017.s009.tiff]

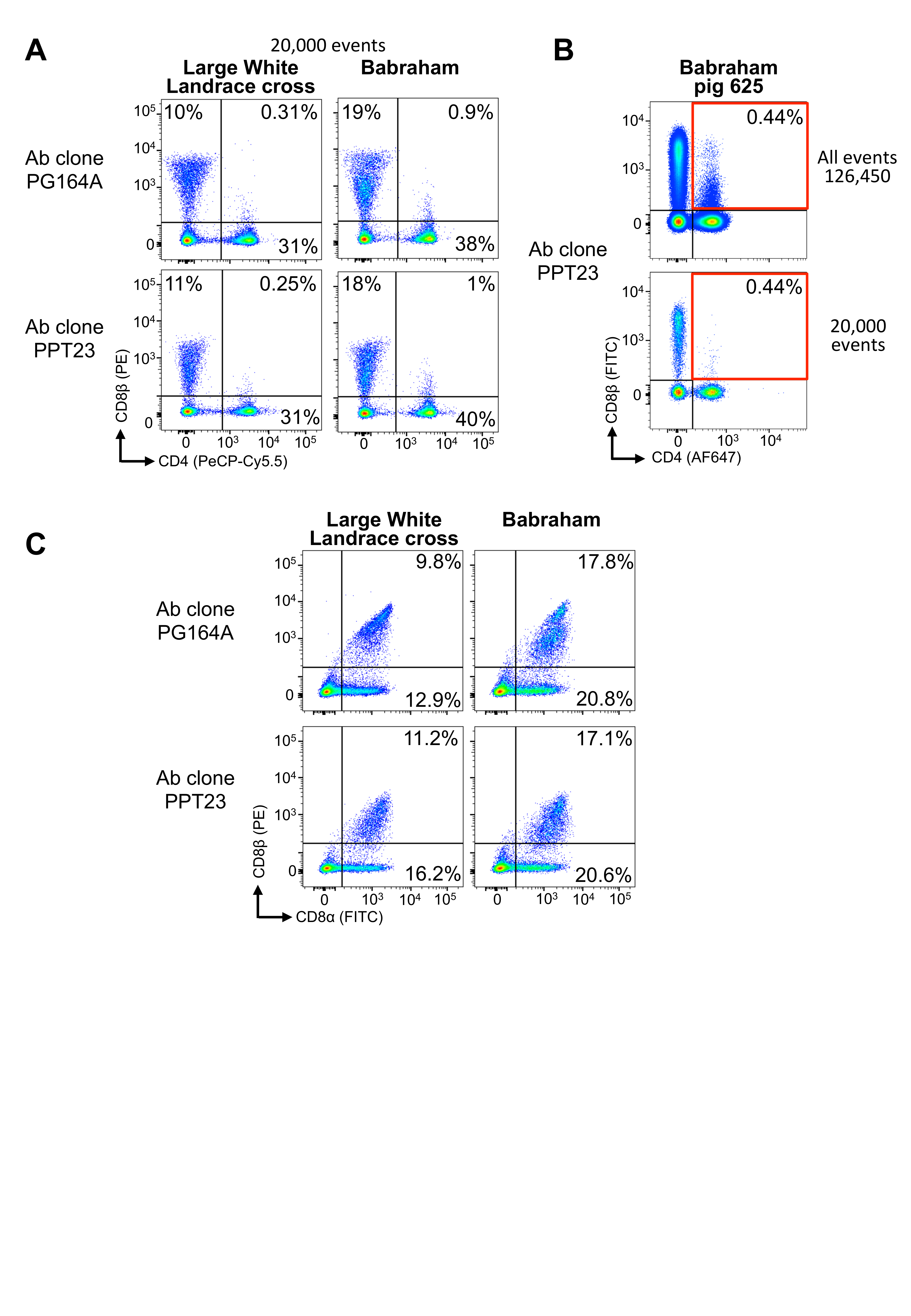

Supplement: S4 Fig — (A) Antibody (Ab) clones PG164A and PPT23 specific for pig cytotoxic T-cells (bind CD8β) were tested on a Large White/Landrace cross and Babraham pig. Cells were gated on viable CD3+ T-cells and displayed as CD8β versus CD4 expression. Percentages are shown for the CD8β+, CD8β+/CD4+ and CD4+ populations. Similar proportions of cells were stained for each of the antibody clones. (B) The CD8β+/CD4+ population ranged between 0.04–1.25% of viable CD3+ T-cells, with a mean of 0.41%. Representative plots from an experimental Babraham pig (625) showing all events, as needed for tetramer analysis, and the same number of events (20,000) as the plots in A. (C) Using the same approach as in A, displaying CD8β versus CD8α staining. Both anti-CD8β antibody clones clearly stained CD8β+/CD8α+ T-cells that were distinct from CD8βneg/CD8α+ T-cells. Percentages are shown for CD8β+/CD8α+ and CD8βneg/CD8α+ populations. (TIFF) [file ppat.1007017.s010.tiff]

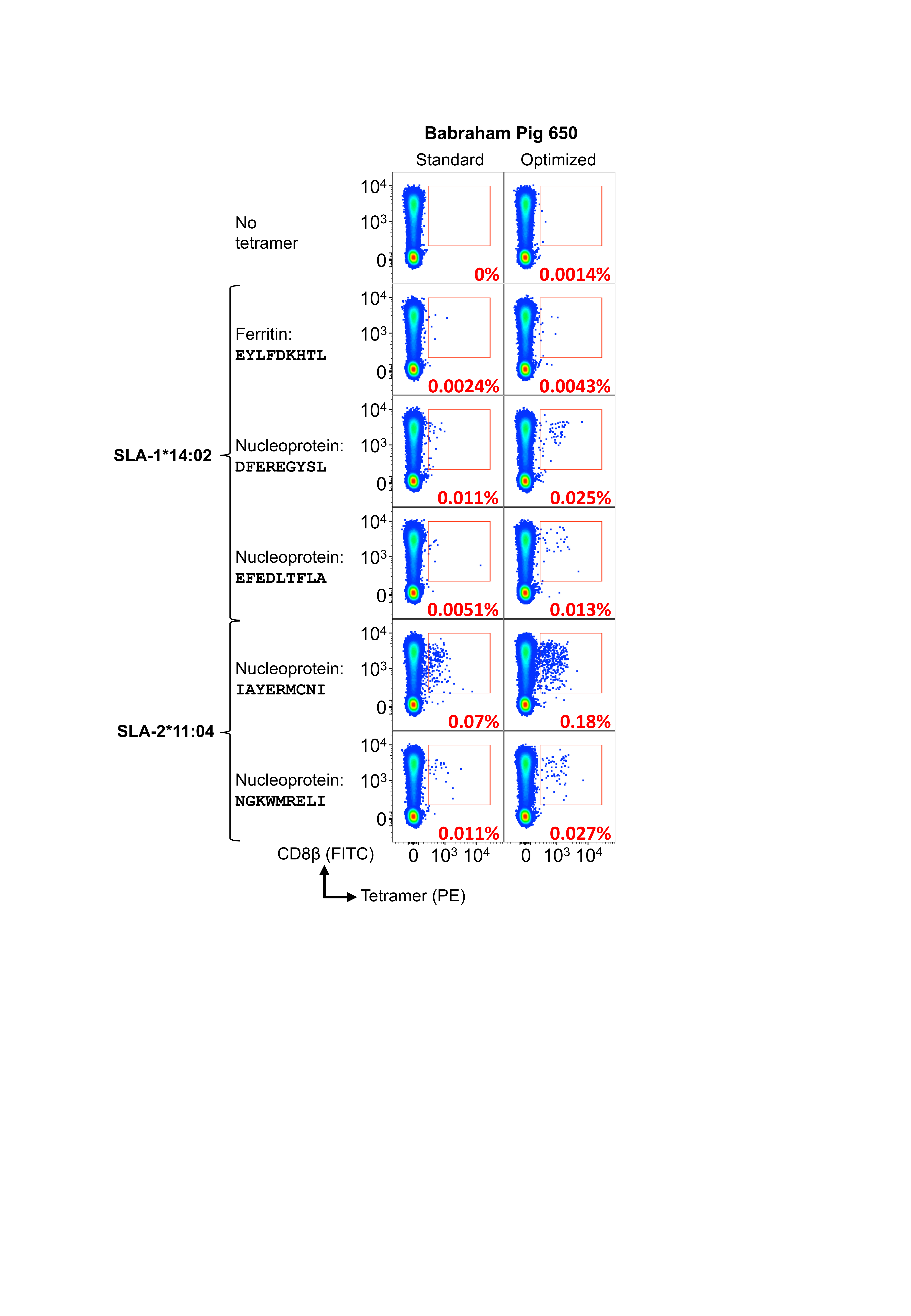

Supplement: S5 Fig — Nucleoprotein or irrelevant peptide SLA tetramers (PE conjugated) were used to stain peripheral blood mononuclear cells from Babraham pig 650, either without (standard protocol) or with (optimized protocol) the addition of protein kinase inhibitor Dasatinib and anti-fluorochrome-PE antibody. The sequences of the nucleoprotein peptides and their restriction are shown. A self-eluted peptide derived from ferritin (EYLFDKHTL) was used as an irrelevant tetramer. The percentage of tetramer+ cells of CD8β+ cells is displayed in red. Gating strategy: lymphocytes, single cells, viability (Vividneg)/CD3+/CD14neg then CD8β+/CD4+ and displayed as CD8β versus tetramer (S1 Fig). (TIFF) [file ppat.1007017.s011.tiff]

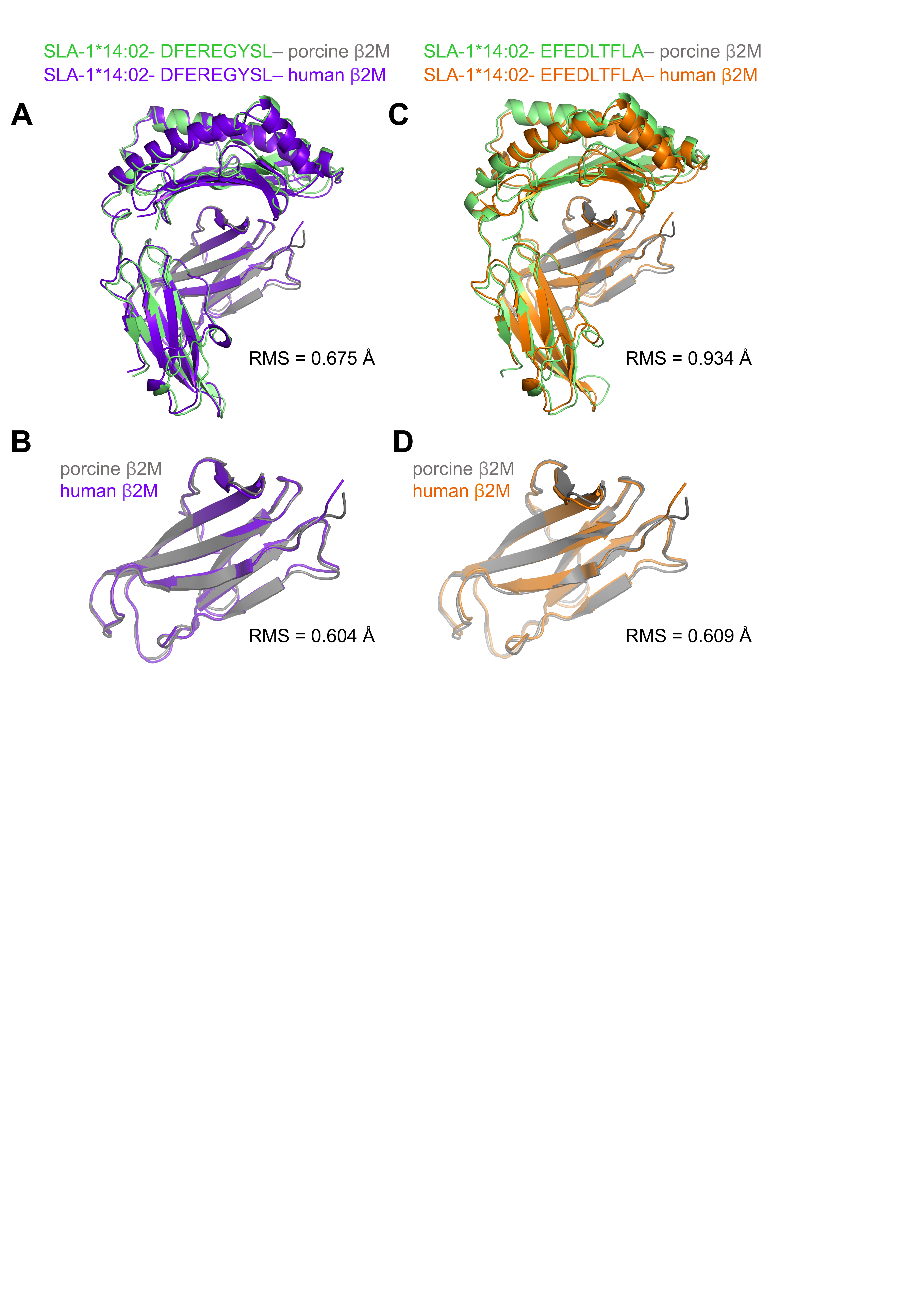

Supplement: S6 Fig — (A) The overall structure of SLA-1*14:02 binding nucleoprotein peptide DFEREGYSL refolded with either porcine β2M (green and grey) or human β2M (purple). (B) Porcine and human β2M from A compared only. (C) The overall structure of SLA-1*14:02 binding nucleoprotein peptide EFEDLTFLA refolded with either porcine β2M (green and grey) or human β2M (orange). (D) Porcine and human β2M from C compared only. Root-means-square (RMS) deviations are displayed for each comparison. (TIFF) [file ppat.1007017.s012.tiff]

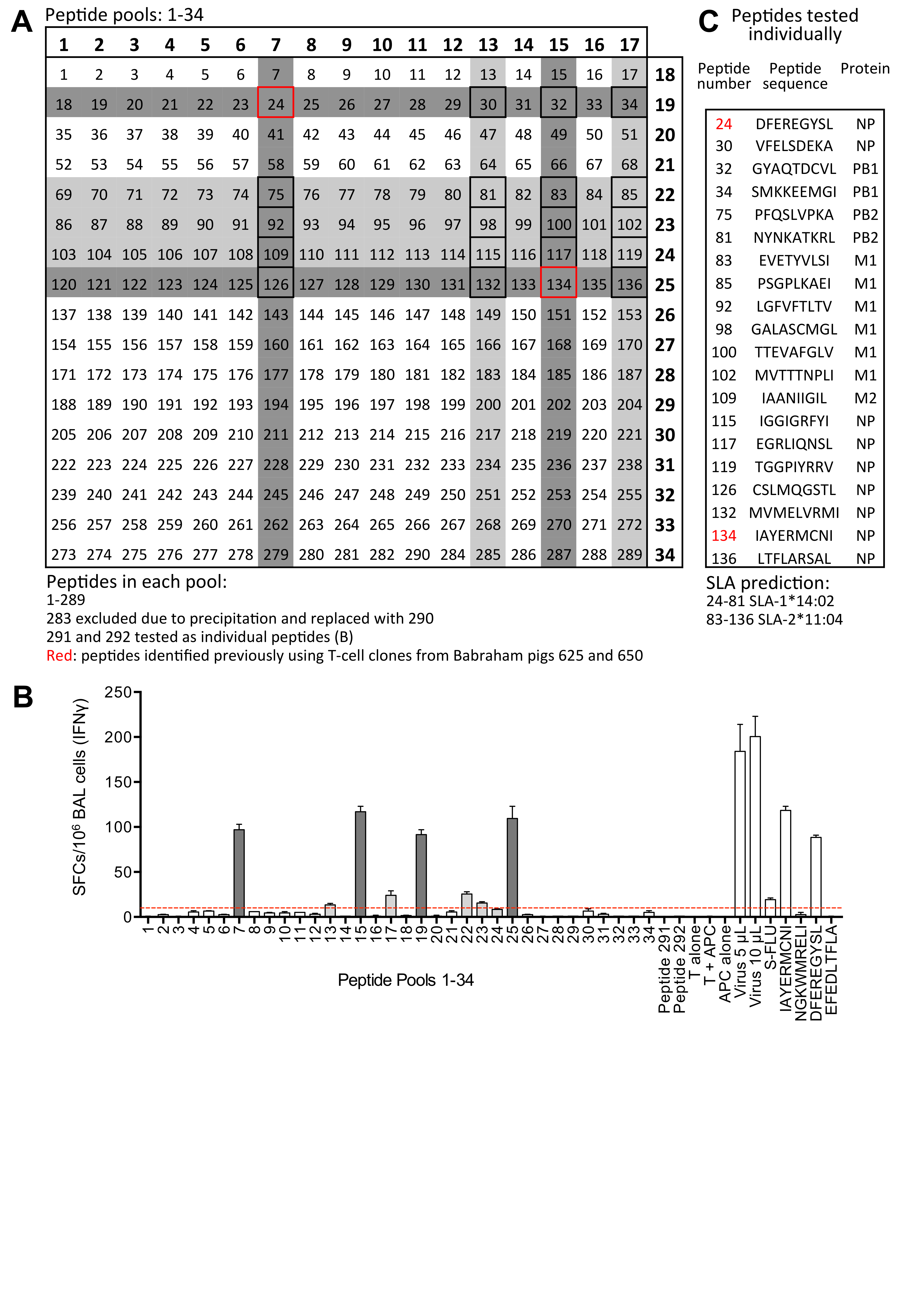

Supplement: S7 Fig — (A) A peptide pool matrix comprising predicted SLA-1*14:02 and SLA-2*11:04 peptides from nucleoprotein (NP), Matrix (M) 1 and 2, and polymerase basic proteins (PB) 1 and 2 (peptide sequences present in S6 Table). Each peptide was present in 2 of the pools allowing rapid ex vivo screening of all peptides with a limited number of cells. Peptides IAYERMCNI, NGKWMRELI, DFEREGYSL and EFEDLTFLA defined in previous experiments were also amongst the matrix. Dark grey indicates a strong ELISPOT response (B) to the respective pool whereas the light grey is for relatively lower responses (B). The intersect between peptide pools of the matrix that elicited a response indicated the individual peptides (boxed) (C) to be tested on Babraham samples (Fig 9). The restricting SLA for each of the selected peptides is shown. (TIFF) [file ppat.1007017.s013.tiff]
